# Metaanalysis of Repetitive Transcranial Magnetic Stimulation (rTMS) Efficacy for OCD Treatment: The Impact of Stimulation Parameters, Symptom Subtype and rTMS-Induced Electrical Field

**DOI:** 10.3390/jcm13185358

**Published:** 2024-09-10

**Authors:** Fateme Dehghani-Arani, Reza Kazemi, Amir-Homayun Hallajian, Sepehr Sima, Samaneh Boutimaz, Sepideh Hedayati, Saba Koushamoghadam, Razieh Safarifard, Mohammad Ali Salehinejad

**Affiliations:** 1 Faculty of Psychology and Educational Sciences, University of Tehran, Tehran 1417935840, Iransbutimaz@ut.ac.ir (S.B.);; 2 Faculty of Entrepreneurship, University of Tehran, Tehran 1738953355, Iran; rezakazemi@ut.ac.ir; 3 Donders Institute for Brain, Cognition, and Behaviour, Radboud University, 6525 Nijmegen, The Netherlands; 4 School of Cognitive Sciences, Institute for Research in Fundamental Sciences, Tehran 1956836613, Iran; 5 Department of Psychology and Neuroscience, University of North Carolina, Chapel Hill, NC 27599, USA; sepidehh@ad.unc.edu; 6 Department of Clinical Psychology, School of Behavioral Sciences and Mental Health, Iran University of Medical Sciences, Tehran 1445613111, Iran; koushamoghadam.s@iums.ac.ir; 7 Department of Psychology and Neurosciences, Leibniz Research Centre for Working Environment and Human Factors, 44139 Dortmund, Germany

**Keywords:** rTMS, OCD, Y-BOCS, systematic review, meta-analysis, electrical field modeling

## Abstract

**Background**: Repetitive transcranial magnetic stimulation (rTMS) has recently demonstrated significant potential in treating obsessive-compulsive disorder (OCD). However, its effectiveness depends on various parameters, including stimulation parameters, OCD subtypes and electrical fields (EFs) induced by rTMS in targeted brain regions that are less studied. **Methods**: Using the PRISMA approach, we examined 27 randomized control trials (RCTs) conducted from 1985 to 2024 using rTMS for the treatment of OCD and conducted several meta-analyses to investigate the role of rTMS parameters, including the EFs induced by each rTMS protocol, and OCD subtypes on treatment efficacy. **Results**: A significant, medium effect size was found, favoring active rTMS (g_PPC_ = 0.59, *p* < 0.0001), which was larger for the obsession subscale. Both supplementary motor area (SMA) rTMS (g_PPC_ = 0.82, *p* = 0.048) and bilateral dorsolateral prefrontal cortex (DLPFC) rTMS (gPPC = 1.14, *p* = 0.04) demonstrated large effect sizes, while the right DLPFC showed a significant moderate effect size for reducing OCD severity (g_PPC_ = 0.63, *p* = 0.012). These protocols induced the largest EFs in dorsal cognitive, ventral cognitive and sensorimotor circuits. rTMS protocols targeting DLPFC produced the strongest electrical fields in cognitive circuits, while pre-supplementary motor area (pre-SMA) and orbitofrontal cortex (OFC) rTMS protocols induced larger fields in regions linked to emotional and affective processing in addition to cognitive circuits. The pre-SMA rTMS modulated more circuits involved in OCD pathophysiology—sensorimotor, cognitive, affective, and frontolimbic—with larger electrical fields than the other protocols. **Conclusions**: While rTMS shows moderate overall clinical efficacy, protocols targeting ventral and dorsal cognitive and sensorimotor circuits demonstrate the highest potential. The pre-SMA rTMS appears to induce electrical fields in more circuits relevant to OCD pathophysiology.

## 1. Introduction

Repetitive transcranial magnetic stimulation (rTMS) is a noninvasive brain stimulation (NIBS) technique with therapeutic application in neuropsychiatric disorders [1,2] and a safety profile [3,4]. In 2018, rTMS was approved by the US Food and Drug Administration (FDA) for the treatment of obsessive compulsive disorder (OCD) [5]. The approved protocol applied high-frequency (HF) deep stimulation to the medial prefrontal and anterior cingulate cortices (ACCs), which has been shown to improve the symptoms of OCD patients [6,7]. Although several meta-analyses focusing on the efficacy of rTMS in OCD have been published [8,9,10,11,12,13], knowledge about optimal stimulation parameters such as frequency and target regions and effective stimulation protocols is not conclusive. This lack of convergence is also reported in all three recent meta-analyses [9,11,14]. In the meta-analysis of Liang et al. [14], low-frequency (LF) stimulation of the supplementary motor area (SMA) and dorsolateral prefrontal cortex (DLPFC) was effective for reducing OCD symptoms in line with other NIBS techniques [15], while the FDA-approved HF-rTMS of anterior cingulate cortex-medial prefrontal cortex (ACC-mPFC) did not show efficacy. In another meta-analysis, the effect size for bilateral DLPFC stimulation was reported to be greater for both HF and LF stimulation compared with other stimulation protocols [11]. In recent meta-analyses, bilateral DLPFC HF rTMS, bilateral SMA LF stimulation, right DLPFC LF stimulation, and HF and LF rTMS of bilateral medial prefrontal cortex/anterior cingulate cortex (mPFC/ACC) were all equally effective at reducing OCD symptoms [9,16].

In addition to different analytical approaches of previous meta-analyses, heterogeneous neural correlates of OCD pathophysiology contribute to the current varied findings regarding rTMS efficacy. For example, the efficacy of LF stimulation of the right DLPFC with regard to reducing OCD symptoms is explained by diminishing activity in the right prefrontal regions of affected patients [14]. In contrast, the efficacy of HF rTMS of the DLPFC is explained by referring to DLPFC hypoactivity in OCD [11]. It seems that various etiological explanations for OCD have resulted in a lack of consensus on effective interventions; further research into new models of OCD pathology may clarify this ambiguity.

A proposed model of OCD etiology identifies five neural circuits involved in symptom generation: ventral cognitive, ventral affective, dorsal cognitive, frontolimbic, and sensorimotor [17,18,19]. These circuits are linked to different OCD symptoms: the frontolimbic circuit relates to intolerance of ambiguity, the dorsal cognitive circuit relates to executive dysfunction, the ventral cognitive circuit relates to impaired response inhibition, the ventral affective system is associated with reward system dysfunction, and the sensorimotor circuit relates to sensory phenomena. Considering different OCD subtypes and symptoms, along with their distinct neural circuits, are therefore crucial for predicting rTMS efficacy in OCD, similar to their role in depression [20,21]. In this line, neuroimaging research has indicated that various neural pathways correspond to different OCD subtypes [22,23]. For instance, individuals with checking obsessions exhibit increased functional connectivity in the motor cortex, while those with washing obsessions show increased connectivity in the anterior insula and orbitofrontal cortex [23].

Furthermore, modeling the electrical fields induced by rTMS in the brain and their effects on circuits involved in OCD can enhance our understanding of various protocols and the heterogeneity observed in earlier meta-analyses. The electrical fields generated by rTMS and other NIBS techniques relate to stimulation strength and brain tissue modulation beneath the target area, both of which are crucial for behavioral change [24,25,26]. This type of modeling also provides insights into dose–response relationships in the brain, and such insights are important for evaluating and refining brain targeting and dosing strategies [27]. No previous metaanalyses have examined the impact of different OCD subtypes and protocol-induced electrical fields, while this approach can provide valuable insights into the efficacy of rTMS for OCD and might explain contradictory findings.

Accordingly, this meta-analysis aims to investigate the efficacy of rTMS studies by examining the neural correlates of OCD subtypes and the effects of stimulation protocols on target regions by modeling the electrical current induced in relevant brain regions by various rTMS protocols. This is the first rTMS meta-analysis to examine the effectiveness of rTMS with respect to symptom subtypes and the electrical fields generated by the protocols. We also examine the impact of stimulation parameters (frequency, treatment duration, and number of pulses) and key aspects of patients’ clinical profiles and study designs on the clinical efficacy of rTMS in treating OCD.

## 2. Methods

We adopted the PRISMA 2020 checklist to carry out the systematic review and meta-analysis [28]. The study protocol is registered with PROSPERO (no: CRD42021257150) and has been updated to focus on OCD subtypes and rTMS-induced electrical fields, addressing novel and unexplored factors.

### 2.1. Search Strategy

A database search was conducted in Scopus, PubMed/MEDLINE, EMBASE, ISI Web of Knowledge, PsycINFO, and ProQuest from 1 January 1985 to 23 July 2024 with no language restrictions. Relevant dissertations on ProQuest, secondary, and conference papers on Scopus were also searched as sources of gray literature. We further updated our search once on 8th of February 2022 and again on 23 July 2024 to include related studies published until July 2024 in PubMed. We also manually searched reference lists of pertinent reviews and meta-analyses. The literature search was performed independently by two authors (S.S and S.B). The following search terms were used in all of the mentioned databases: (“Transcranial Magnetic Stimulation” OR “TMS” OR “rTMS” OR “Repetitive Transcranial Magnetic Stimulation”) AND (“Obsessive Compulsive Disorder” OR “Obsessive-Compulsive Disorder” OR “OCD” OR “Compulsion” OR “Obsession” OR “Obsessive Compulsive Neurosis” OR “Obsessive Compulsive Neuroses” OR “Obsessive-Compulsive Neurosis” OR “Obsessive-Compulsive Neuroses” OR (“Disorder” AND “Obsessive Compulsive”) OR (“Disorder” AND “Obsessive-Compulsive”) OR (“Neurosis” AND “Obsessive Compulsive”) OR (“Neurosis” AND “Obsessive-Compulsive”) OR (“Neuroses” AND “Obsessive Compulsive”) OR (“Neuroses” AND “Obsessive-Compulsive”).

### 2.2. Selection Process

Automation tools were used to implement the exclusion criteria, and the following categories were excluded: news, books, reviews, editorials, case studies, and literature reviews. As for the selection process, two authors (S.S and S.B) independently screened the included studies and two authors (S.K and S.H) independently reviewed the full text of the screened studies and selected those that met the eligibility criteria. Any complications in the selection process were resolved by the third author (R.S).

### 2.3. Data Collection Process

Data were extracted from selected studies using an Excel sheet created by S.S., and then verified by A.H. In cases of disagreement, the second author (RK) was consulted. When data were not available in the full text, we either requested data directly from the authors via email or used a graph digitizer service (http://getdata-graph-digitizer.com/) to extract information from graphs. The required information included (1) meta-data (authors’ names, year of publication); (2) demographics (sample size per group, gender, age); (3) disorder characteristics (baseline Yale–Brown Obsessive Compulsive Scale score, comorbid MDD, treatment resistance, illness duration); (4) medications; (5) rTMS parameters and intervention protocol (stimulation frequency, intensity, total pulses per session, number of sessions, brain targets); (6) research methods (sham stimulation technique; blinding); (7) mean and standard deviation of post-intervention and follow-up Y-BOCS scores (total and subscales, if available) for both groups; (8) response rate criteria.

### 2.4. Eligibility Criteria

**Figure 1:**
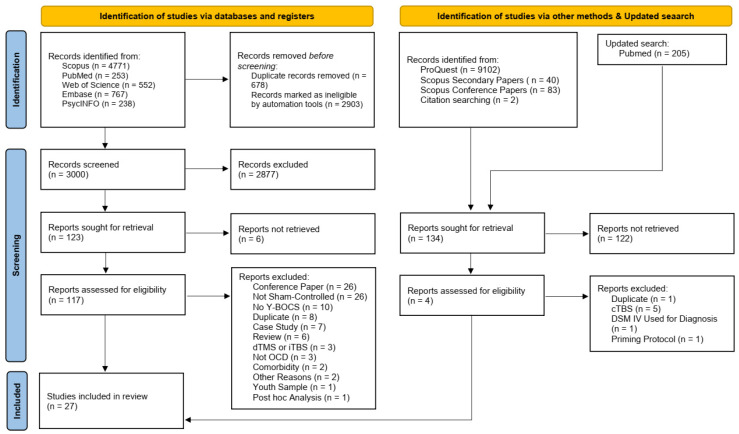
Flowchart diagram of the study selection process.

The inclusion criteria comprised (1) randomized controlled trials (RCTs) involving adults (age > 18) diagnosed with OCD according to the DSM-V or ICD-10, without any comorbid psychiatric conditions except for Major Depressive Disorder (MDD) or anxiety. (2) Studies needed to provide the necessary data for effect size estimation in their full texts or have such data available upon request from the authors. (3) Only studies employing rTMS were selected, and (4) OCD symptom severity had to be assessed using the Yale–Brown Obsessive Compulsive Scale (Y-BOCS). Exclusions included case reports, case series, reviews, studies lacking a sham control group, those with fewer than ten treatment sessions, and those utilizing deep rTMS (drTMS) or theta burst stimulation. The study selection process is illustrated in Figure 1.

### 2.5. Risk of Bias Assessment and Quality of Evidence

The risk of bias in each study was assessed according to the Cochrane tool for assessing the risk of bias in randomized trials [29] in six domains, and the assessments were conducted independently by S.S and A.H. When a specific category was not clearly defined in the study methods, the study category was listed as unclear. The last author (M.A.S) resolved any complications in the final rating, if needed. A.H assessed the quality of evidence for our main outcome measure (Y-BOCS) in all studies and for the cortical target in subgroup analyses, using the Criteria for Recommendations Assessment, Development, and Evaluation (GRADE) [30].

### 2.6. Statistical Analysis

All the statistical analyses were conducted in R using *dmetar*, *metafor*, and *meta packages* (version 4.1.2; R Core Team, 2022) [31]. The Metafor package was used to compute the Standardized Mean Difference (SMD) [32], the meta package was used for the forest plot and pooling effect sizes, and the *dmetar* package was used for sensitivity analysis and assessing publication bias [33,34]. We used the method proposed by [35] for computing the SMDs of studies with a pretest–posttest control group design (PPC). This method estimates a bias-corrected form of Hedge’s g for pretest–posttest control group design (g_PPC_). The primary outcome was the pre- to post-stimulation YBOCS total score change. The SMD and variance for each study were computed according to Morris and the *escalc* function of *metafor* package, assuming that there was an r = 0.5 pretest–posttest correlation (metafor-project.org/doku.php/analyses:morris2008). To ensure that this correlation value did not lead to a biased result, we conducted a sensitivity analysis using r = 0.3 and r = 0.7 correlations as well (the results are reported in the Supplementary Materials).

Due to expected clinical and between-study heterogeneity, a random-effects model was used to pool effect sizes. The random effects model was fitted using restricted maximum likelihood (REML). Also, we used Knapp–Hartung adjustments [36]) to calculate the %95 confidence interval around the pooled effect. Furthermore, heterogeneity was assessed by Cochran’s Q, $\tau^{2}$ and $I^{2}$ in each condition. $I^{2}$ values above 50% were considered to be evidence of high between-study heterogeneity. Publication bias was assessed visually utilizing funnel plots, and the Egger test [37] was also conducted to confirm the conclusions. We also used the P-cure method [38] to investigate the evidential value of studies effects’. Using this method, we could assess whether our meta-analysis data reveal a true effect, and estimate the effect’s size. Additionally, this method explicitly controls for questionable research practices like p-hacking. Additionally, using *find.outliers* and *InfluenceAnalysis* functions from *dmetar* package, we ran a sensitivity analysis and verified the robustness of our pooled effect by removing the influential studies. The code and data necessary for replication are available via the link below: https://osf.io/6vq7d/.

### 2.7. Electrical Field Modeling of rTMS Studies

**Figure 2:**
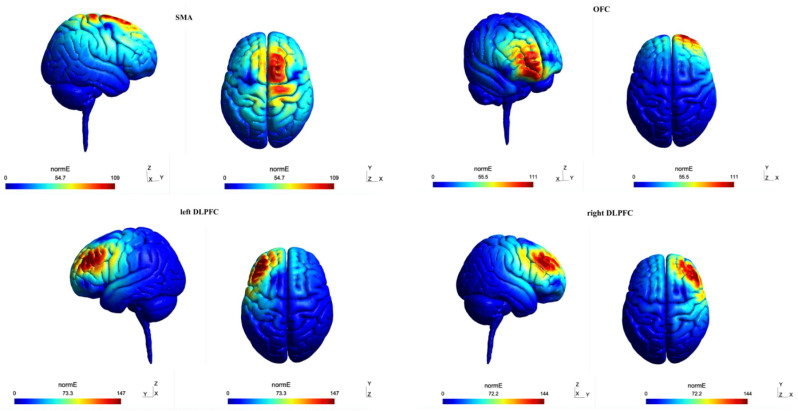
The distribution of the induced normed electrical field for each rTMS protocol.

The electrical field models are simulated using SimNIBS 3.2 software for each of the cortical targets (right and left DLPFC, SMA, and orbitofrontal-OFC) according to our meta-analysis [39] using the approach described in previous studies [40]. SimNIBS creates a volume conductor model by segmenting a structural MRI image (weighted T1 or T2) to simulate the induced electric fields. The simulation was performed on the standard unbiased MNI head model provided by simNIBS. The default electrical conductivity values were chosen for the simulation (scalp: 0.465 S/m, bone: 0.01 S/m; cerebrospinal fluid: 1.654 S/m; gray matter: 0.275 S/m; white matter: 0.126 S/m). In addition, we set the scalp–coil distance to 4mm and since the $\frac{dI}{dt}$ values vary among different manufacturers [41], for comparison’s sake, we set this value to $10^{8}\;A/s$ (Figure 2).

To investigate the variability of results among OCD subtypes, we calculated the induced electrical field (norm E) across various neurocircuits related to OCD neurobiology [17,18,19]. These circuits included the sensorimotor circuit, dorsal and ventral cognitive circuit, ventral affective circuit, and frontal-limbic circuit. The core regions and their respective MNIs are listed in Table S1, and include the sensorimotor circuit (SMA [42], posterior putamen [43], primary motor cortex [44], somatosensory cortex [45], insula [46], and thalamus [47]); dorsal cognitive circuit (pre-SMA [42], DLPFC [48], dorsomedial PFC [49], dorsal caudate [50], and thalamus [47]); ventral cognitive circuit (inferior frontal gyrus [42], ventrolateral PFC [51], ventral caudate [50], and thalamus [47]); ventral affective circuit (OFC [43], nucleus accumbens [50], and thalamus [47]); and frontolimbic circuit (ventromedial PFC [52] and amygdala [53]). Results from the simNIBS simulation for each cortical target region were imported into Matlab (MathWorks, 2019), where we wrote custom code to compute the induced electrical field averaged over a 10mm diameter sphere centered on each MNI coordinate.

## 3. Results

A thorough review of the literature search results revealed 27 studies [54,55,56,57,58,59,60,61,62,63,64,65,66,67,68,69,70,71,72,73,74,75,76,77,78,79,80] with 784 participants (428 in the active stimulation group and 356 in the sham group) included in the meta-analysis. The average age of participants was 33.86 for the active group and 33.83 for the sham group. Table 1 presents the key characteristics of the studies examining the effects of rTMS on OCD included in the meta-analysis.

### 3.1. Efficacy of rTMS in Reducing OCD Symptoms Severity

**Figure 3:**
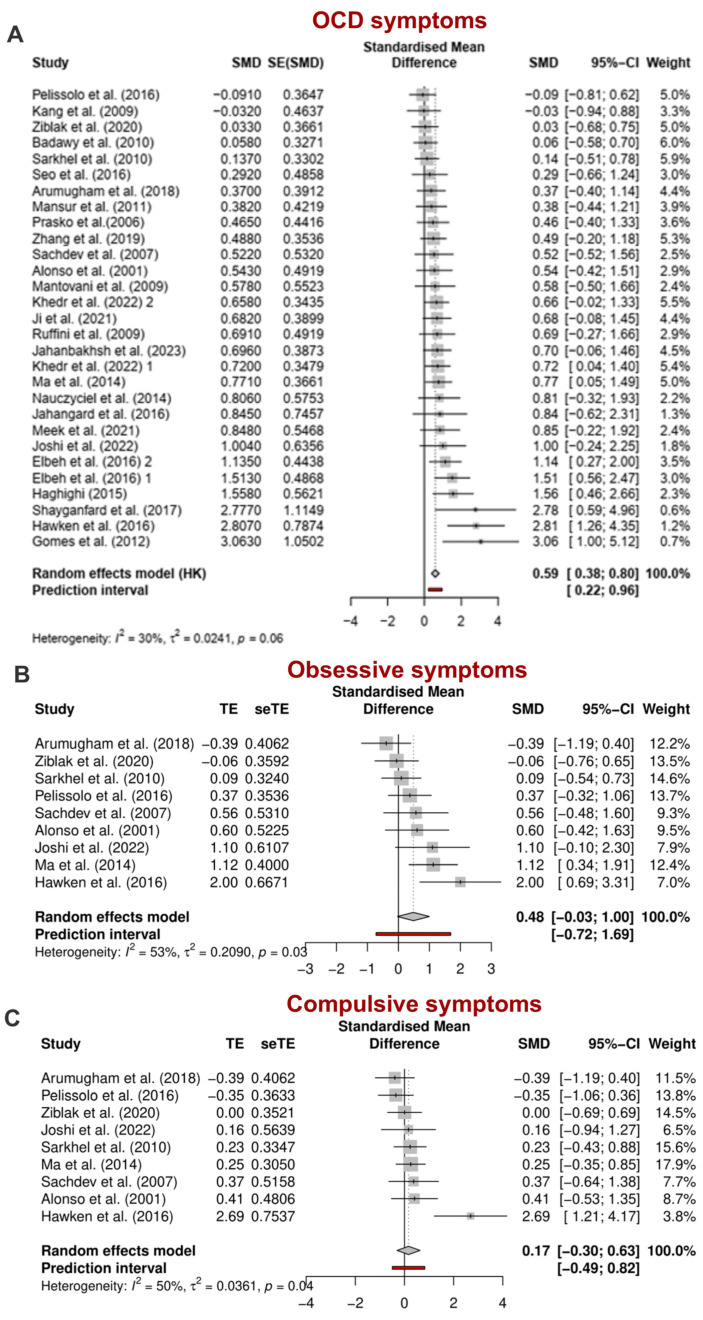
(**A**) Pooled effect sizes (g_ppc_) of rTMS studies for reducing OCD symptoms. (**B**) Pooled effect sizes (gppc) of rTMS studies for reducing obsession symptoms (**C**) Pooled effect sizes (g_ppc_) of rTMS studies for reducing compulsion symptoms, CI: confidence interval, SMD: standardized mean difference [54,55,56,57,58,59,60,61,62,63,64,65,66,67,68,69,70,71,72,73,74,75,76,77,78,79,80].

In total, 27 studies were examined to investigate the primary outcome, which was the change in the scores of Y-BOCS before and after rTMS treatment. g_PPC_ was used as the measure of the effect size, which was 0.59 overall. The results showed that active rTMS is significantly more effective than sham treatment for OCD [g_PPC_ = 0.59, 95% CI = 0.38; 80, *p* < 0.0001]. The between-study heterogeneity was estimated at τ^2^ = 0.02 [95% CI = 0.00; 0.58], with an I^2^ value of 30%, as shown in the forest plot in Figure 3A. The prediction interval ranged was [0.22;0.96] indicating that we expect a positive effect from future studies. To ensure that the analysis results were not predominantly influenced by a single study, a sensitivity analysis was conducted. This analysis revealed that the overall findings were significantly impacted by the studies of Gomes et al. (2012) [63], Hawken et al. (2016) [68], and Shayganfard et al. (2017) [72]. Even when excluding these studies, rTMS demonstrated significant effectiveness on OCD symptoms [gPPC = 0.52, 95% CI = 0.36; 0.68, *p* < 0.0001]. Heterogeneity estimates decreased substantially after removing the outlier studies [τ^2^ = 0.00, 95% CI = 0.00; 0.12, I^2^ = 0.00%] (Figure S2).

To investigate the effects of rTMS on obsessive and compulsive symptoms separately, we conducted a meta-analysis of studies with available Y-BOCS subscale data (K studies = 9). The estimated effect size for the obsession subscale was higher [gPPC = 0.48, 95% CI = −0.03; 1.00, *p* = 0.06] than for the compulsion subscale [gPPC = 0.17, 95% CI = −0.30; 0.66, *p* = 0.44]. The between-study heterogeneity was τ^2^ = 0.21 [95% CI = 0.00; 1.64] for obsessions and τ^2^ = 0.03 [95% CI = 0.00; 2.59] for compulsions, with I^2^ values of 53% and 50%, respectively. The prediction intervals ranged from [−0.72 to 1.69] for obsessions and [−0.49; 0.82] for compulsions, indicating that negative intervention effects cannot be ruled out in future studies (Figure 3B,C). Due to the limited number of available studies, we did not perform a sensitivity analysis on the subscales.

### 3.2. Subgroup Analyses

**Figure 4:**
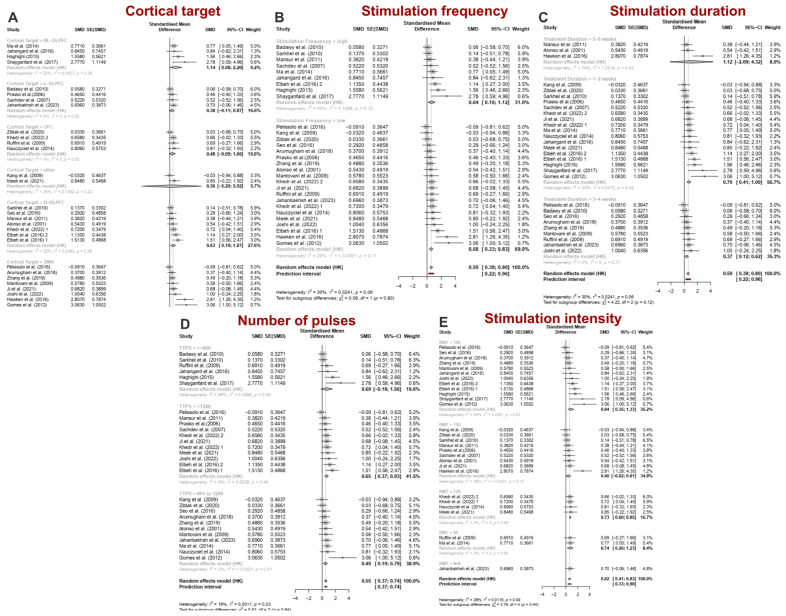
(**A**) Pooled effect sizes (gppc) of rTMS studies for reducing OCD symptoms based on the cortical target of rTMS, BL: bilateral, L: left, R: right, DLPFC: dorsolateral prefrontal cortex, OFC: orbitofrontal cortex, SMA: supplementary motor area. (**B**) Effect sizes (gppc) for OCD symptoms based on the frequency of rTMS. (**C**) Pooled effect sizes (gppc) for OCD symptoms based on the duration of rTMS treatment. (**D**) Pooled effect sizes (gppc) for OCD symptoms based on the total induced pulses of rTMS per session, TTPS: total pulse per session. (**E**) Effect sizes (gppc) for OCD symptoms based on the intensity of rTMS, RMT: resting motor threshold, CI: confidence interval, SMD: standardized mean difference [54,55,56,57,58,59,60,61,62,63,64,65,66,67,68,69,70,71,72,73,74,75,76,77,78,79,80].

**Figure 5:**
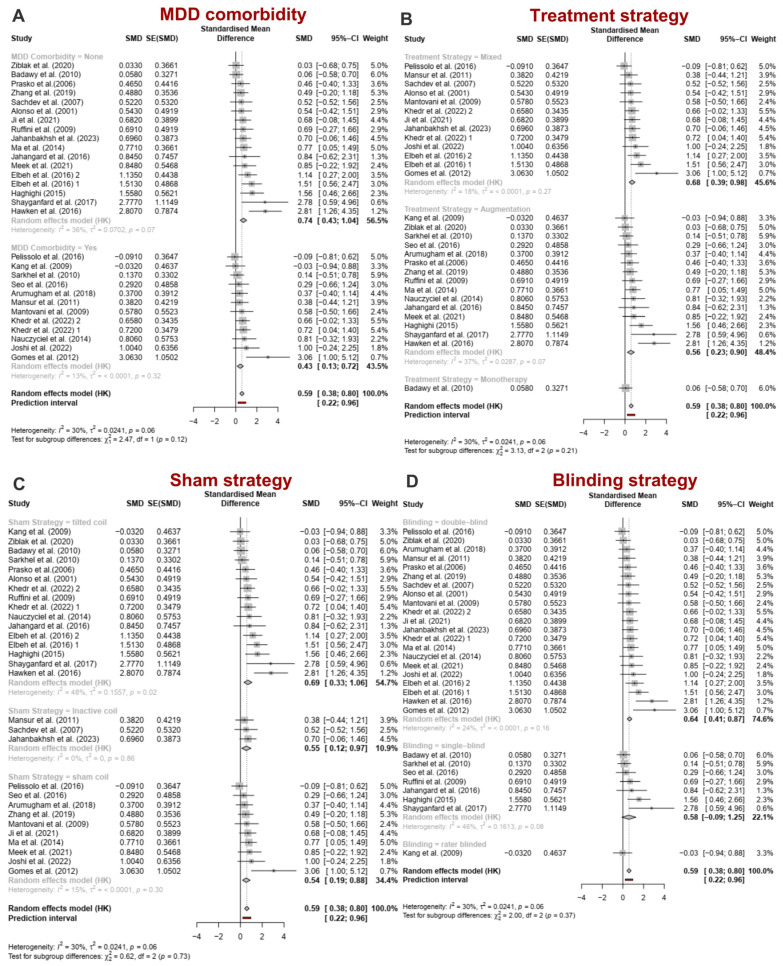
(**A**) Effect sizes (gppc) for OCD symptoms based on the presence of MDD comorbidity. (**B**) Effect sizes (gppc) for OCD symptoms based on the strategy of rTMS treatment. (**C**) Effect sizes (gppc) for OCD symptoms based on the sham stimulation strategy. (**D**) Effect sizes (gppc) for OCD symptoms based on the blinding strategy. MDD: major depressive disorder, CI: confidence interval, SMD: standardized mean difference [54,55,56,57,58,59,60,61,62,63,64,65,66,67,68,69,70,71,72,73,74,75,76,77,78,79,80].

Subgroup analyses were performed based on stimulation parameters, including cortical target, stimulation frequency, treatment duration, number of pulses, intensity (Figure 4A–E), clinical factors such as comorbidity with Major Depressive Disorder (Figure 5A,B), and influential factors related to the methodology of the sham treatments and the blinding strategy (Figure 5C,D).

#### 3.2.1. Stimulation-Related Parameters

*Cortical Target*. The included studies were categorized into six subgroups based on the cortical target: bilateral DLPFC (k = 4), unilateral right DLPFC (R-DLPFC) (k = 7), unilateral left DLPFC (L-DLPFC) (k = 4), SMA (k = 8), OFC (k = 4), and other areas (k = 2). Significant improvements in OCD symptoms were observed in the SMA [gPPC = 0.82, 95% CI = 0.01; 1.62, *p* = 0.048], bilateral DLPFC [gPPC = 1.14, 95% CI = 0.08; 2.20, *p* = 0.04], and R-DLPFC [gPPC = 0.63, 95% CI = 0.19; 1.07, *p* = 0.012] subgroups. In contrast, no significant effects of rTMS were found for OFC [gPPC = 0.48, 95% CI = −0.09; 1.06, *p* = 0.076], L-DLPFC [gPPC = 0.38, 95% CI = −0.11; 0.87, *p* = 0.088], and other cortical regions [gPPC = 0.36, 95% CI = −5.2; 5.92, *p* = 0.561]. The bilateral DLPFC subgroup exhibited the largest effect size, while the SMA subgroup showed the greatest heterogeneity [τ^2^_SMA_ = 0.41, I^2^_SMA_ = 60.8%] (Figure 4A). Excluding outliers reduced effect size and heterogeneity for both SMA and bilateral DLPFC subgroups, rendering the effect size for bilateral DLPFC non-significant [SMA: gPPC = 0.42, 95% CI = 0.06; 0.78, *p* = 0.03, τ^2^ = 0.00, I^2^ = 0.00%; bilateral DLPFC: gPPC = 0.98, 95% CI = −0.04; 2.01, *p* = 0.054, τ^2^ = 0.00, I^2^ = 0.00%] (Figure S3).

*Frequency of stimulation*. Both HF-rTMS (k = 9) and LF-rTMS (k = 20) significantly outperformed sham stimulation, leading to significant improvements in symptoms. Notable reductions in Y-BOCS scores were recorded for HF-rTMS (gPPC = 0.64, 95% CI = 0.16; 1.12, *p* = 0.015) and LF-rTMS (gPPC = 0.58, 95% CI = 0.33; 0.83, *p* < 0.0001). The heterogeneity between studies was τ^2^ = 0.11 for HF-rTMS and τ^2^ < 0.0001 for LF-rTMS, with I^2^ values of 40.5% and 28.7%, respectively (Figure 4B). After removing outlier studies, effect sizes remained significant (HF-rTMS: gPPC = 0.57, *p* = 0.015; LF-rTMS: gPPC = 0.52, *p* < 0.0001), and heterogeneity decreased significantly (HF-rTMS: I^2^ = 25.8%, τ^2^ = 0.07; LF-rTMS: I^2^ = 0.00%, τ^2^ = 0.00) (Figure S4).

*Treatment duration*. Significant changes were observed compared to the sham group after 1 to 2 weeks of rTMS (k = 17; gPPC = 0.70, 95% CI = 0.41; 1.00, *p* < 0.001) and 3 to 4 weeks (k = 9; gPPC = 0.37, 95% CI = 0.12; 0.62, *p* < 0.01) (Figure 4C), with a greater effect size in the 1- to 2-week period. In contrast, rTMS administered for over 4 weeks (5 to 6 weeks, k = 3) showed no significant changes (gPPC = 1.12, 95% CI = −2.09; 4.32, *p* = 0.273). The respective results without the outliers are shown in Figure S5.

*Number of pulses*. Significant improvements were observed for pulse counts between 800 and 1200 per session (k = 11; gPPC = 0.49, 95% CI = 0.19; 0.79, *p* < 0.01) and for counts exceeding 1200 (k = 11 studies; gPPC = 0.65, 95% CI = 0.37; 0.93, *p* < 0.001), but not for fewer than 800 pulses/session (k = 6; gPPC = 0.69, 95% CI = −0.18; 1.56; *p* = 0.095). The largest effect size was for 1200 pulses/session (Figure 4D). Sensitivity analysis did not lead to significant changes in the estimates of interest (Figure S6).

*Intensity of stimulation*. Significant changes were observed with stimulation at 80% (k = 2; gPPC = 0.74, 95% CI = 0.26; 1.23, *p* = 0.032), 100% (k = 12; gPPC = 0.84, 95% CI = 0.35; 1.33, *p* = 0.003), and 120% (k = 4; gPPC = 0.73, 95% CI = 0.60; 0.85, *p* < 0.001) of the RMT, but not at 110% (k = 9; gPPC = 0.40, 95% CI = −0.02; 0.81, *p* = 0.06) (Figure 4E). Notably, the stimulation at 100% had a slightly larger effect size than at 120%. After sensitivity analysis, 110% RMT became statistically significant (k = 8; gPPC = 0.31, 95% CI = 0.09; 0.53, *p* = 0.012) (Figure S7).

#### 3.2.2. Clinical and Design-Related Factors

*MDD comorbidity*: The absence of MDD comorbidity significantly influenced OCD symptoms (K = 17, gPPC = 0.74, 95% CI = 0.43; 1.04; *p* < 0.001), while the presence of MDD comorbidity did not result in significant changes in OCD symptoms (k = 12, gPPC = 0.43, 95% CI = 0.13; 0.72; *p* < 0.01) (Figure 5A). After removing outliers, the effect sizes for all subgroups became significant: MDD present (gPPC = 0.39, 95% CI = 0.17; 0.61) and MDD absent (gPPC = 0.64, 95% CI = 0.40; 0.88) (Figure S8).

Treatment strategy: Complementary treatment (K = 15, gPPC = 0.56, 95% CI = 0.23; 0.9, *p* = 0.003) and mixed treatment (K = 13, gPPC = 0.68, 95% CI = 0.39; 0.98, *p* < 0.001) resulted in significant changes, while rTMS treatment alone (K = 1, gPPC = 0.06, 95% CI = −0.58; 0.7, *p* = 0.86) did not (Figure 5B). The sensitivity analysis outcomes are detailed in the Supplementary Materials (Figure S9).

Sham strategy and blinding: Sham stimulation methods show that using sham coils (k = 10, gPPC = 0.54, 95% CI = 0.19; 0.88, *p* = 0.007) and tilted coils (k = 16, gPPC = 0.69, 95% CI = 0.33; 1.06, *p* = 0.001) are the most effective strategies for administering sham rTMS. Among the three blinding methods (double-blind, single-blind, and evaluator blinding), the double-blind method is the most effective (k = 18; gPPC = 0.64, 95% CI = 0.41; 0.87, *p* < 0.001) (Figure 5C,D).

### 3.3. Results of Electric Field Modeling of Four Stimulation Protocols

The results of computational modeling of electrical fields induced by rTMS protocols show that applying rTMS to the pre-SMA generates an average electric field of 12.86 ± 16.11 V/m across the whole brain. The strongest electrical fields were induced in the pre-SMA (41.47 V/m), somatosensory cortex (41.03 V/m), ventrolateral prefrontal cortex (21.29 V/m), ventromedial prefrontal cortex (16.19 V/m), and orbitofrontal cortex (11.25 V/m) respectively, which correspond to the dorsal cognitive, sensorimotor, ventral cognitive, frontolimbic, and ventral affective circuits, respectively.

Next, our results show that rTMS over the left DLPFC generates an average electric field of 13.37 ± 16.52 V/m across the whole brain. The highest average electric fields from this protocol are induced in the DLPFC (57.64 V/m), ventrolateral prefrontal cortex (25.41 V/m), primary motor area (21.1 V/m), nucleus accumbens (9.88 V/m), and ventromedial prefrontal cortex (8.91 V/m) respectively, corresponding to the dorsal cognitive, ventral cognitive, sensorimotor, ventral affective, and frontolimbic circuits.

Applying rTMS to the right DLPFC generates an average electric field of 13.08 ± 16.48 V/m across the whole brain, with the strongest field observed in the DLPFC (55.48 V/m), followed by the inferior frontal gyrus (38.49 V/m), primary motor cortex (21.55 V/m), nucleus accumbens (9.97 V/m), and ventromedial prefrontal cortex (6.21 V/m), corresponding to the dorsal cognitive, ventral cognitive, sensorimotor, ventral affective, and frontolimbic circuits, respectively.

Lastly, rTMS applied to the OFC produced an average electric field of 11.48 ± 12.29 V/m across the brain, and electrical fields of 35.08 V/m in the DLPFC, 27.07 V/m in the ventrolateral prefrontal cortex, 12.03 V/m in the ventromedial prefrontal cortex, and 11.46 V/m in the orbitofrontal cortex, reflecting activity in the dorsal cognitive, ventral cognitive, and frontolimbic circuits (Table S2).

In summary, rTMS protocols targeting the right and left DLPFC generated the strongest electrical fields in cognitive circuits (lateral and medial PFC), while pre-SMA and OFC rTMS protocols produced larger electrical fields in regions associated with emotional and affective processing. The pre-SMA rTMS was the only protocol that modulated the most involved circuits (sensorimotor, cognitive, affective, and frontolimbic circuits) with larger electrical fields compared to other protocols.

### 3.4. Publication Bias

To address the influence of unpublished negative results (missing studies) and small-study effects, we performed publication bias analyses using a funnel plot, Egger’s regression, and a P-curve. The data revealed an asymmetrical pattern in the funnel plot, suggesting potential publication bias. To further explore the relationship of this pattern with statistical significance, we created a contour-enhanced funnel plot [81] (Figure S10), which indicated that three small studies showed significant effects despite having high standard errors; these studies were included in the sensitivity analysis. We conducted Egger’s regression analysis to quantify the asymmetry in the funnel plot (t = 5.061, *p* < 0.001). The trim and fill method adjusted the funnel plot’s asymmetry by adding nine studies (Figure S11) and modified the effect size (gPPC = 0.41, 95% CI = 0.23; 0.59, *p* < 0.0001), which was lower than the estimated effect size before the sensitivity analysis (gPPC = 0.59, 95% CI = 0.38; 0.80, *p* < 0.0001).

Another method to estimate publication bias in this study was the P-curve method. This method was developed to cover the shortcomings of the trim and fill method. Seven studies from the meta-analysis were considered in the final p-curve analysis. The distribution of observed *p*-values is shown in Figure S12. The results of the P-curve analysis showed that the distribution of *p*-values was skewed (Z = −2.51, *p* = 0.006), which is indicative of the evidential value of the studies. Also, the distribution of the *p*-values was not flatter (Z = 0.53, *p* = 0.70) and this indicates that the studies were not underpowered. Also, *p*-curve’s estimate of the true effect size was d = 0.66 which was comparatively larger than the estimated effect size (g_PPC_ = 0.59) (the *p*-curve analysis results are reported in Table S3).

### 3.5. Risk of Bias Assessment

**Figure 6:**
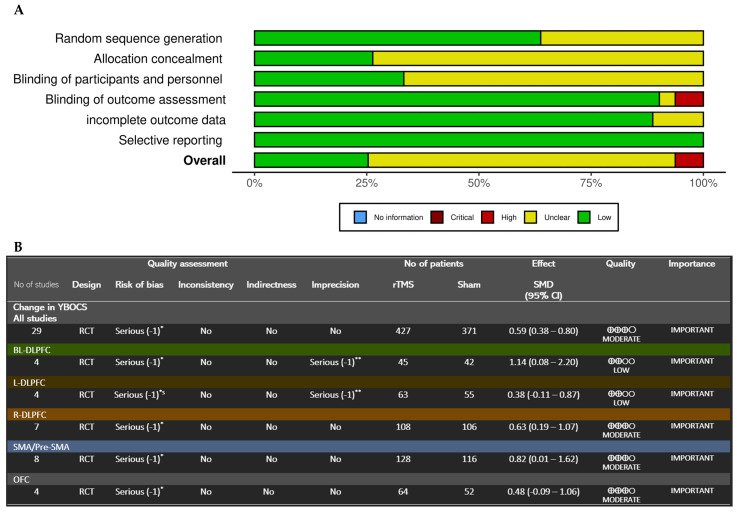
(**A**) Bar plot showing the distribution of risk-of-bias judgments across bias domains. The bars indicate the proportion of studies within each domain, providing an overview of the collective bias risk. The colors represent: low risk (green), some concerns (yellow), and high risk (red). (**B**) GRADE assessment results. *: Lack of Intention-to-treat analysis in several studies; many didn’t report the allocation concealment procedure (Only 6 studies had done and intention-to-treat analysis 4 of which are in the SMA/pre-SMA group). In addition, the funnel plot shows an asymmetrical pattern suggesting the presence of publication bias. **: 95% CI has broad intervals or/and includes both significant benefit of treatment and notable harm.

The Cochrane RoB instrument indicated that 26% of studies had a low risk of bias, 70% had an unclear risk, and 4% had a high risk. There was no evidence of selective reporting across studies; however, the primary source of unclear risk was the allocation concealment procedure, which occurred in 81% of the included studies (see Figure 6A). Risk of bias assessment for each study is reported in Supplementary Figure S1.

### 3.6. Quality of Evidence

The GRADE framework evaluation indicated that the evidence quality for rTMS’s effectiveness in reducing OCD symptoms was moderate across all studies. However, the evidence was of low quality for studies targeting the orbitofrontal cortex (OFC), while it remained moderate for other cortical targets (Figure 6B).

## 4. Discussion

This was the first meta-analysis of rTMS randomized controlled trials in OCD focusing on behavioral effects, symptom-specific effects, and computational modeling of neural effects of commonly used rTMS protocols in OCD severity and its subtypes (obsession and compulsion). A significant moderate effect of rTMS, regardless of protocol, was found. All three rTMS protocols with significant effect sizes for reducing OCD severity (bilateral DLPFC rTMS, SMA rTMS, right DLPFC rTMS) induce the highest level of electric fields in both sensorimotor and ventral cognitive circuits. Furthermore, rTMS was more effective in reducing obsession than compulsion symptoms. The pre-SMA rTMS induced electrical fields in all OCD-pathophysiology circuits (sensorimotor, cognitive, affective, and frontolimbic circuits) compared to other protocols. In what follows, we discuss the response to rTMS in OCD patients with respect to target region and induced electrical fields, symptom subtype, and stimulation parameters (e.g., session number, number of pulses, stimulation intensity).

### 4.1. Efficacy of rTMS in OCD: Target Region

Bilateral DLPFC rTMS, SMA rTMS, and right DLPFC rTMS significantly reduced OCD symptoms in the active group compared to the sham group, with bilateral DLPFC stimulation yielding the greatest symptom reductions. Our computational modeling further showed that DLPFC stimulation in both hemispheres generated the highest electric field in dorsal and ventral cognitive circuits. This, along with the regions of the circuits with the strongest electrical fields (e.g., lateral PFC), suggests that enhancing the executive control system [82,83] might explain the effectiveness of DLPFC rTMS in reducing OCD severity. For example, dysfunction in the ventral cognitive circuit is linked to impaired response inhibition in OCD [84,85,86,87]. This is broadly related to the enhancement of a goal-directed system over the habit system [88]. The efficacy of DLPFC rTMS aligns with other NIBS studies in disorders with executive dysfunction, demonstrating that increased activity in the lateral PFC is linked to improved executive function and reduced symptoms [89,90,91,92,93,94,95,96].

rTMS of the SMA produces the strongest electric fields in dorsal cognitive and sensorimotor circuits, as well as in the frontolimbic and ventral affective systems, compared to other protocols. The pre-SMA and sensorimotor cortex interact extensively with the putamen and are involved in motor behavior and habit systems [88]. Compulsions are typically thought to stem from obsessions; however, some arise from discomfort, such as physical sensations or feelings of incompleteness, known as sensory phenomena [97,98]. In OCD patients, the presence and severity of SP correlate with symptoms of order and symmetry [99] and are linked to increased gray matter volume in sensorimotor areas [45]. Additionally, the sensorimotor circuit plays a role in generating SP and habit formation in OCD, creating a cycle that perpetuates symptom generation and maintenance [84]. Therefore, the primary mechanism of SMA rTMS can be described as regulating the exaggerated habit system in OCD. This protocol involved key cognitive correlates associated with dorsal and ventral cognitive circuits. Dysfunction in these circuits impairs working memory, emotion regulation, and planning [84]. Executive function, which is essential for goal-directed behavior [100] is compromised in OCD patients [82,83]. Although it may not directly cause specific symptoms, it likely exacerbates OCD symptoms and is linked to dysfunction in the dorsal cognitive circuit [19,84].

### 4.2. Efficacy of rTMS in OCD: Symptoms Subtypes

Subtypes of OCD and related disorders (e.g., ADHD) might be associated with distinct neuropsychological and pathophysiological profiles [101,102,103]. We found that rTMS is more effective at reducing obsessions than compulsions, although the results were not statistically significant. Of 26 studies analyzed, only 9 provided subtype data, including 4 targeting the DLPFC, 4 targeting the SMA, and 1 targeting the OFC. The reduction in obsessions is assumed to result from rTMS’s modulation of the dorsal cognitive circuit, while the DLPFC protocols also affected the ventral circuit. Fear-based obsessions are linked to frontolimbic hyperactivity [84] and dorsal cognitive hypoactivity, indicating that both circuits may contribute to mental obsessions. The idea about the subtype-specific effect of rTMS intervention should be interpreted cautiously due to controversies and the limited specificity of OCD subtypes [104,105]. While some patients may exhibit predominant symptoms in recognized OCD subtypes, a combination of symptoms are often present. Additionally, there is significant overlap between OCD pathophysiology and other disorders, [106,107] with some suggesting the existence of new subtypes, such as OCD with urinary obsessions [108].

### 4.3. Efficacy of rTMS in OCD: Stimulation Parameters

#### 4.3.1. Number of rTMS Sessions and Number of Pulses

An interesting finding of this meta-analysis is about treatment duration for reducing OCD symptoms. Increasing the number of sessions or extending treatment beyond four weeks did not significantly alleviate OCD symptoms. In this context, a recent meta-analysis of rTMS studies in OCD found that fewer sessions were linked to a better treatment response, but this result lost significance after statistical corrections [9]. Consistent with our study, a recent meta-analysis on the effects of rTMS for various psychiatric disorders, including OCD, indicated that 10–20 rTMS sessions are adequate for therapeutic response, with more sessions providing no added benefit [109,110]. One possible explanation relates to the total number of pulses received by the patient. In our study, more than 800 pulses were linked to therapeutic response, while 1200 pulses yielded a greater effect size. A study examining two groups with 600 and 1200 pulses at a frequency of 5 Hz found that a higher number of pulses per session does not necessarily lead to more significant changes in cortical excitability [111]. Consequently, increasing the number of sessions to deliver more pulses may not lead to a greater therapeutic response, despite previous studies [8] indicating a positive correlation between pulse quantity and therapeutic efficacy. Other factors, such as a ceiling effect and the method of pulse delivery, may also play crucial roles in this relationship [112]. It is noteworthy that the optimal number of rTMS treatments and session duration remain uncertain [113] and require more studies in the future.

#### 4.3.2. Delivering rTMS as the Sole Intervention or as Augmentation

Our results indicate that rTMS, when used as an augmentation or in a mixed treatment approach, significantly outperforms monotherapy in reducing symptoms, with the mixed approach demonstrating a larger effect size. However, since only one study employed a monotherapy approach, these findings should be interpreted with caution. The combined treatment strategy has gained attention in depression tretament, particularly through the combination of rTMS and psychotherapy [114]. Psychotherapeutic interventions like cognitive behavioral therapy influence the frontolimbic circuit, and their combination with rTMS can enhance therapeutic outcomes [115]. However, the belief that combined interventions always produce a superior synergistic effect is misleading. Another promising but less studied approach in NIBS for OCD treatment is the combination of rTMS with dopaminergic and serotonergic medications, which play a role in OCD pathophysiology and may enhance NIBS-induced neuroplasticity [116,117,118]. The timing of the interventions and their influence on brain physiology must also be taken into account. Here is also important to consider the time of day and participants’ sleep pressure during the intervention as these factors greatly affect cortical excitability and brain stimulation-induced neuroplasticity [119,120].

### 4.4. Efficacy of rTMS in OCD: Protocol-Induced Electrical Field

Our computational modeling of common rTMS protocols shows that all protocols significantly affect three circuits related to OCD pathophysiology (dorsal and ventral cognitive and sensorimotor circuits) along two other important circuits, namely the frontolimbic and ventral affective circuits; however, to a lesser degree. Protocols targeting the right and left DLPFC generated the strongest electrical fields in cognitive circuits (lateral and medial PFC), while pre-SMA and OFC protocols had greater effects in regions linked to emotional and affective processing in addition to their larger effects on cognitive circuits. The electrical field induced by pre-SMA rTMS protocol involved was overall stronger in all involved circuits (sensorimotor, cognitive, affective, and frontolimbic). This suggests that interventions targeting multiple neural circuits related to OCD pathophysiology may be more effective, aligning with the heterogeneous pathophysiology of OCD. Furthermore, the only difference between rTMS over the right DLPFC and the left was that the former elicited a stronger electrical field in the inferior frontal gyrus, a crucial area for response inhibition [121]. Importantly, there is increased functional connectivity between pre-SMA and inferior frontal gyrus, which is associated with motor response inhibition in OCD [42]. This could partially explain the significant therapeutic effects of rTMS on the right DLPFC.

## 5. Conclusions

In conclusion, rTMS studies in OCD demonstrate moderate therapeutic effects. However, efficacy can vary largely based on cortical regions and stimulation parameters, and partly based on OCD subtypes. Protocols that target more neural circuits related to OCD pathophysiology, such as SMA rTMS, bilateral DLPFC, and right DLPFC rTMS, tend to show greater effectiveness. Patients with a predominance of obsessional symptoms may experience more benefit from rTMS, although this requires further research. Additionally, increasing the number of rTMS sessions does not necessarily enhance therapeutic efficacy. This study suggests considering OCD subtypes and using the most relevant protocol tailored to the patient’s pathophysiology, which can be achieved through individualized rTMS based on clinical profiles and neuroimaging data.

## Figures and Tables

**Table 1:** Characteristics of the included studies.

Authors		Active rTMS			Sham rTMS		Blinding	Cortical Target	Frequency (Hz)	Sessions	Total Pulses	Treatment Duration (Weeks)	Follow-Up (Weeks)	Sham Strategy	Treatment Resistant	Comorbid MDD
	*n*	Age (Mean ± SD)	F/M	N	Age (Mean ± SD)	F/M										
Alonso et al. (2001) [54]	10	39.2 ± 13	8/2	8	30.3 ± 9.5	4/4	double-blind	R-DLPFC	1 (LF)	18	21,600	6	10	tilted coil	Yes	None
Prasko et al.(2006) [55]	18	28.9 ± 7.7	5/13	12	33.4 ± 8.7	7/5	double-blind	L-DLPFC	1 (LF)	10	18,000	2	2	tilted coil	Yes	None
Sachdev et al. (2007) [56]	10	29.5 ± 9.9	3/7	8	35.8 ± 8.2	5/3	double-blind	L-DLPFC	10 (HF)	10	15,000	2	N/A	Inactive coil	Yes	None
Ruffini et al. (2009) [57]	16	41.5 ± 9.06	6/10	7	39.3 ± 9.55	3/4	single-blind	L-OFC	1 (LF)	15	9000	3	12	tilted coil	Yes	None
Kang et al. (2009) [58]	10	28.6 ± 12.66	2/8	10	26.2 ± 10.52	1/9	rater blinded	R-DLPFC and SMA	1 (LF)	10	12,000	2	2	tilted coil	Yes	Yes
Sarkhel et al. (2010) [59]	21	29.38 ± 6.55	11/10	21	31.95 ± 7.81	8/13	single-blind	R-DLPFC	10 (HF)	10	8000	2	2	tilted coil	N/A	Yes
Badawy et al. (2010) [60]	20	26 ± 5.58	10/10	20	28.9 ± 5.7	13/7	single-blind	L-DLPFC	20 (HF)	15	12,000	3	N/A	tilted coil	Yes	None
Mantovani et al. (2009) [61]	9	39.7 ± 8.6	4/5	9	39.4 ± 10.2	3/6	double-blind	BL-pre-SMA	1 (LF)	20	24,000	4	N/A	sham coil	Yes	Yes
Mansur et al. (2011) [62]	13	42.1 ± 11.9	6/7	14	39.3 ± 13.9	8/6	double-blind	R-DLPFC	10 (HF)	30	60,000	6	6	Inactive coil	Yes	Yes
Gomes et al. (2012) [63]	12	35.5 ± 7.5	8/4	10	37.5 ± 16	5/5	double-blind	SMA	1 (LF)	10	12,000	2	12	sham coil	Yes	Yes
Ma et al. (2014) [64]	25	27.12 ± 8.97	8/17	21	29.86 ± 9.42	8/13	double-blind	BL-DLPFC	8–12 (HF)	10	6480–8720	2	1	sham coil	Yes	None
Nauczyciel et al. (2014) [65]	9	40	7/2	10	39	8/2	double-blind	OFC	1 (LF)	10	12,000	1	4	tilted coil	Yes	Yes
Haghighi (2015) [66]	10	34.9 ± 5.91	3/7	11	36.55 ± 3.95	6/5	single-blind	BL-DLPFC	20 (HF)	20	7500	2	2	tilted coil	Yes	None
Seo et al. (2016) [67]	14	34.6 ± 9.8	6/8	13	36.3 ± 12.5	7/6	single-blind	R-DLPFC	1 (LF)	15	18,000	3	N/A	sham coil	Yes	Yes
Hawken et al. (2016) [68]	10	33 ± 10	3/7	12	34 ± 14	8/4	double-blind	BL-SMA	1 (LF)	25	N/A	6	6	tilted coil	Yes	None
Jahangard et al. (2016) [69]	5	32.40 ± 8.97	1/4	5	33.80 ± 5.81	2/3	single-blind	BL-DLPFC	20 (HF)	20	7500	2	2	tilted coil	Yes	None
Pelissolo et al. (2016) [70]	20	39.1 ± 10.4	13/7	16	42.3 ± 10.6	9/7	double-blind	pre-SMA	1 (LF)	20	30,000	4	N/A	sham coil	Yes	Yes
Elbeh et al. (2016) 1 [71]	15	26.8 ± 5.2	4/11	15	25.5 ± 4	5/10	double-blind	R-DLPFC	1 (LF)	10	20,000	2	12	tilted coil	Yes	None
Elbeh et al. (2016) 2 [71]	15	28.9 ± 3.9	6/9	15	25.5 ± 4	5/10	double-blind	R-DLPFC	10 (HF)	10	20,000	2	12	tilted coil	Yes	None
Shayganfard et al. (2017) [72]	5	33.8 ± 9.55	4/1	5	33.2 ± 7.86	2/3	single-blind	BL-DLPFC	20 (HF)	10	7500	2	2	tilted coil	Yes	None
Arumugham et al. (2018) [73]	19	27.74 ± 7.88	3/16	17	30.71 ± 10.43	5/12	double-blind	pre-SMA	1 (LF)	18	21,600	3	N/A	sham coil	Yes	Yes
Zhang et al. (2019) [74]	25	32.2 ± 13.25	10/15	24	39.38 ± 17.04	10/14	double-blind	pre-SMA	1 (LF)	20	24,000	4	2	sham coil	Yes	None
Ziblak et al. (2020) [75]	19	41.47 ± 10.23	14/5	15	36.53 ± 13.69	9/6	double-blind	R-OFC	1 (LF)	20	20,000	2	2	tilted coil	Yes	None
Ji et al. (2021) [76]	20	27.75 ± 1.58	5/15	17	27.65 ± 1.73	5/12	double-blind	R-preSMA	1 (LF)	14	25,200	2	N/A	sham coil	Yes	None
Meek et al. (2021) [77]	10	45 ± 16.7	6/4	10	38.3 ± 11.5	4/6	double-blind	dACC	1 (LF)	20	24,000	2	N/A	sham coil	N/A	N/A
Joshi et al. (2022) [78]	13	31.85 ± 7.56	7/6	11	25.36 ± 5.07	3/8	double-blind	SMA	1 (LF)	20	32,000	3	N/A	sham coil	No	Yes
Khedr et al. (2022) 1 [79]	20	36.9 ± 11.5	11/9	20	35.35 ± 9.38	10/10	double-blind	R-DLPFC	1 (LF)	10	15,000	2	12	tilted coil	No	Yes
Khedr et al. (2022) 2 [79]	20	34.05 ± 10.23	11/9	20	35.35 ± 9.38	10/10	double-blind	R- OFC	1 (LF)	10	15,000	2	12	tilted coil	No	Yes
Jahanbakhsh et al. (2023) [80]	15	34.07 ± 8.34	9/6	15	34.53 ± 9.75	11/4	double-blind	L-DLPFC	1 (LF)	15	18,000	3	12	Inactive coil	Yes	None

R-DLPFC: right dorsolateral prefrontal cortex, L-DLPFC: left dorsolateral prefrontal cortex, BL-DLPFC: bilateral dorsolateral prefrontal cortex, pre-SMA: pre-supplementary motor area, dACC: dorsal anterior cingulate cortex, OFC: orbitofrontal cortex, HF: high-frequency stimulation, LF: low-frequency stimulation, N/A: not applicable to studies without follow-up data.

## Data Availability

Data sharing is not applicable to this systematic review and metanalysis article. Code and data for replication are, however, available at the link below: https://osf.io/6vq7d/.

## Supplementary Materials

The following supporting information can be downloaded at https://www.mdpi.com/article/10.3390/jcm13185358/s1, Figure S1: Risk of Bias Evaluation for the included Randomized Controlled Trials; Figure S2: Effect sizes (gppc) for OCD symptoms with outlier studies removed; Figure S3: Effect sizes (gppc) for OCD symptoms based on the cortical target of rTMS with outlier studies removed; Figure S4: Effect sizes (gppc) for OCD symptoms based on the frequency of rTMS with outlier studies removed; Figure S5: Effect sizes (gppc) for OCD symptoms based on the duration of rTMS treatment with outlier studies removed; Figure S6: Effect sizes (gppc) for OCD symptoms based on the total induced pulses of rTMS per session with outlier studies removed; Figure S7: Effect sizes (gppc) for OCD symptoms based on the intensity of rTMS with outlier studies removed; Figure S8: Effect sizes (gppc) for OCD symptoms based on the presence of MDD comorbidity with outlier studies removed; Figure S9: Effect sizes (gppc) for OCD symptoms based on the strategy of rTMS treatment rTMS with outlier studies removed; Figure S10: Contour-enhanced funnel plot for the detection of publication bias; Figure S11: Funnel plot using the trim & fill method; Figure S12: P-curve analysis of 7 statistically significant *p*-values (*p* < 0.05), of which 6 are *p* < 0.025; Table S1: MNI Coordinates for Each ROI Used for rTMS-Induced Electrical Field Simulation [42,43,44,45,46,47,48,49,50,51,52,53]; Table S2: Induced electrical field values for each rTMS protocol corresponding to each ROI and neural circuit; Table S3: *p*-curve analysis details. References [42,43,44,45,46,47,48,49,50,51,52,53] are cited in the Supplementary Materials.
