# Supplementary material for: Metaanalysis of Repetitive Transcranial Magnetic Stimulation (rTMS) Efficacy for OCD Treatment: The Impact of Stimulation Parameters, Symptom Subtype and rTMS-Induced Electrical Field
Source: J Clin Med. 2024 Sep 10;13(18):5358. doi: 10.3390/jcm13185358 (PMC11432318; doi:10.3390/jcm13185358)
Supplement: Supplementary file 1 [file jcm-13-05358-s001.zip › jcm-3132509-supplementary.pdf]

Supplementary Figures and Tables

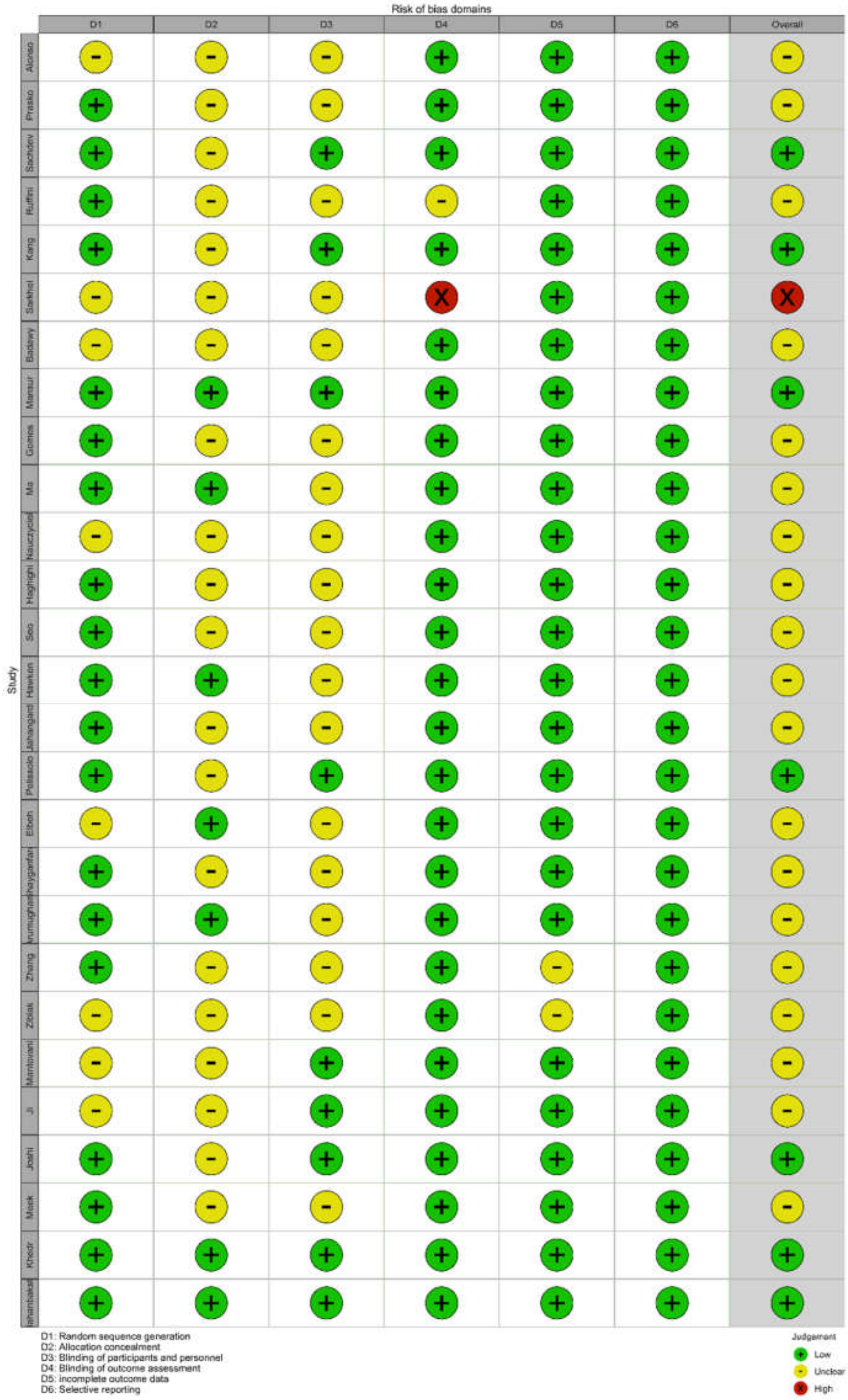

Figure S1. Risk of Bias Evaluation for the included Randomized Controlled Trials. Traffic light plot illustrating domain-specific risk-of-bias assessments for individual study results. Each column represents a different study, and each row corresponds to a specific bias domain.

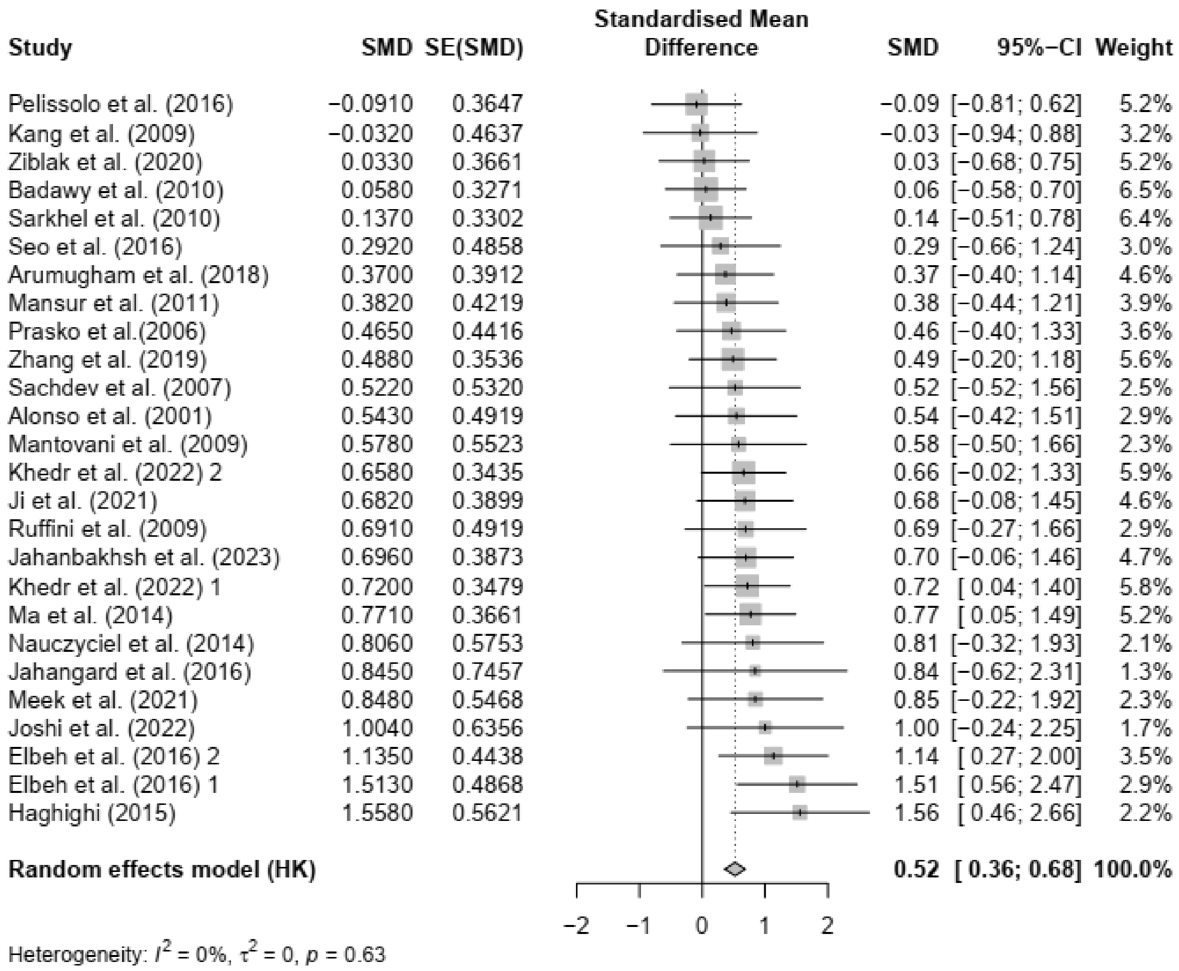

Figure S2: Effect sizes (gppc) for OCD symptoms with outlier studies removed.

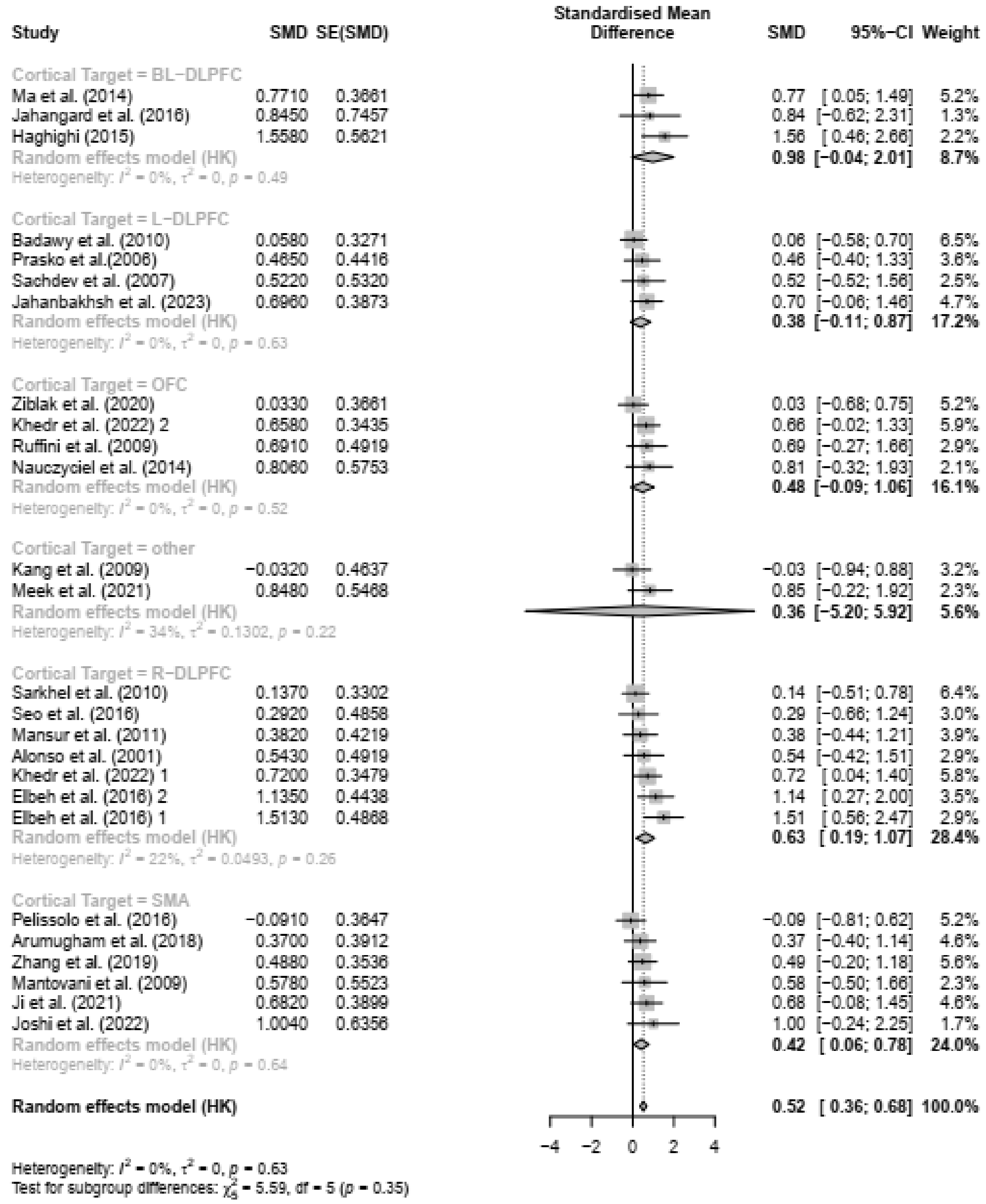

Figure S3. Effect sizes (gppc) for OCD symptoms based on the cortical target of rTMS with outlier studies removed.

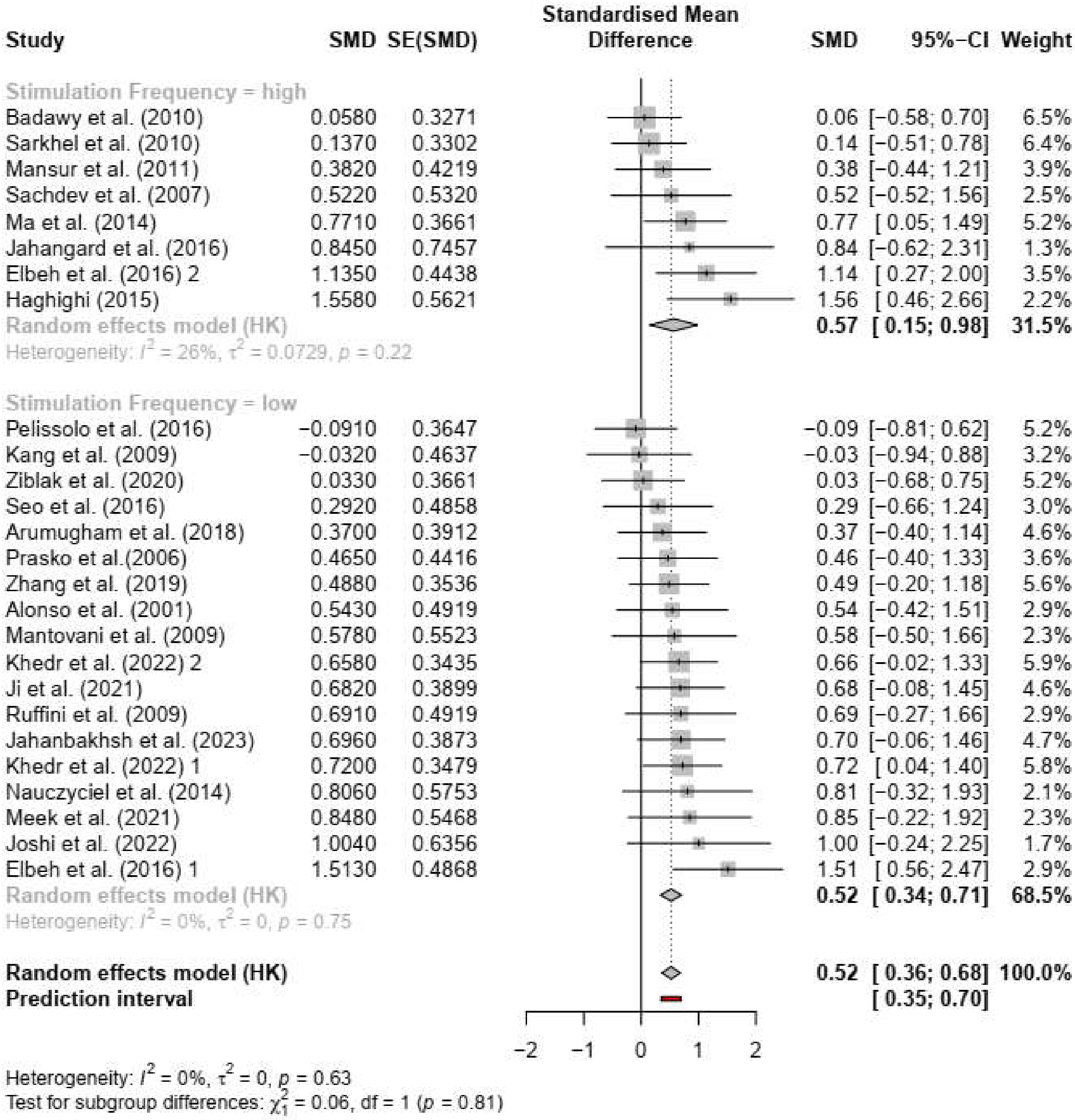

Figure S4. Effect sizes (gppc) for OCD symptoms based on the frequency of rTMS with outlier studies removed.

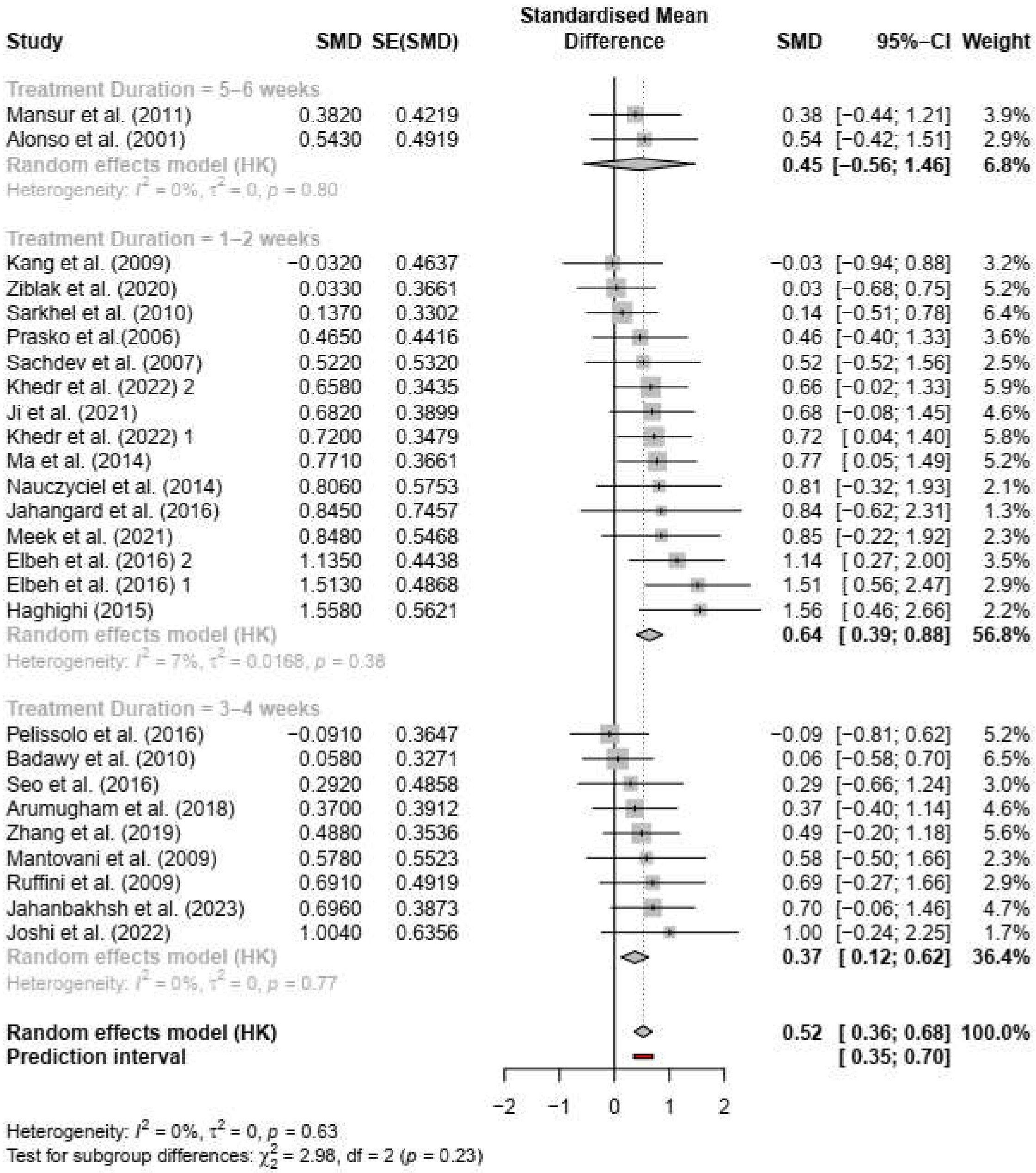

Figure S5. Effect sizes (gppc) for OCD symptoms based on the duration of rTMS treatment with outlier studies removed.

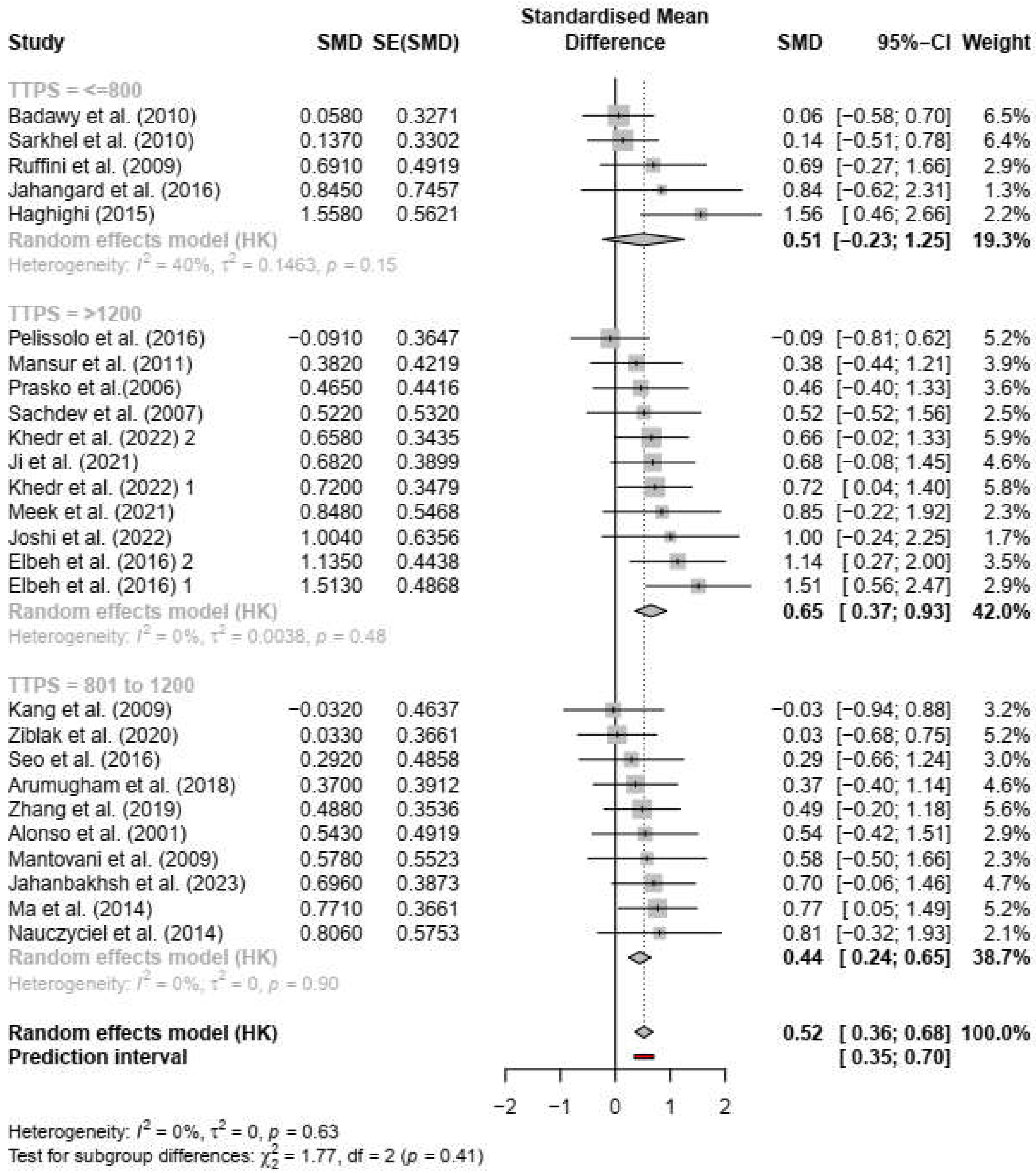

Figure S6. Effect sizes (gppc) for OCD symptoms based on the total induced pulses of rTMS per session with outlier studies removed.

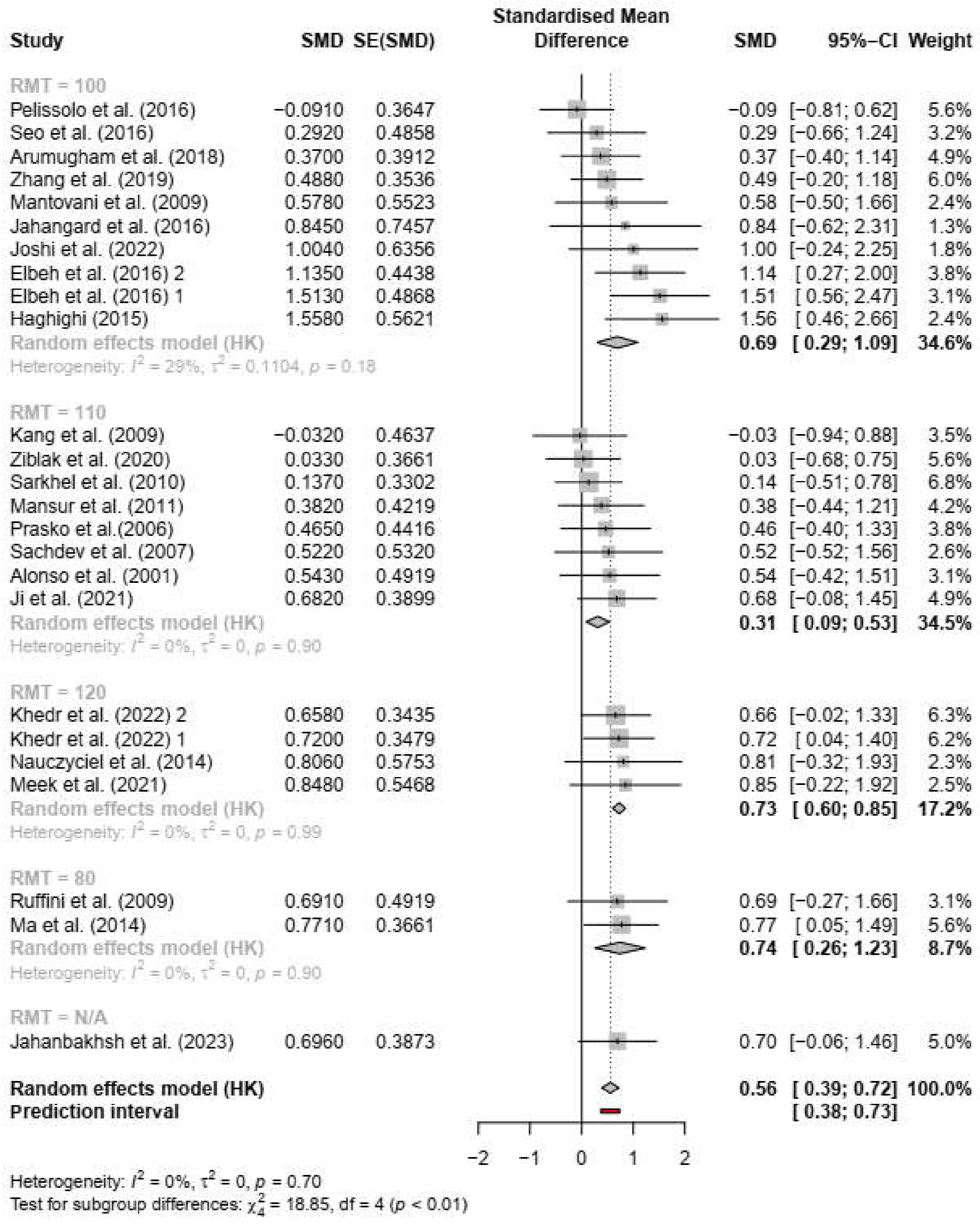

Figure S7. Effect sizes (gppc) for OCD symptoms based on the intensity of rTMS with outlier studies removed.

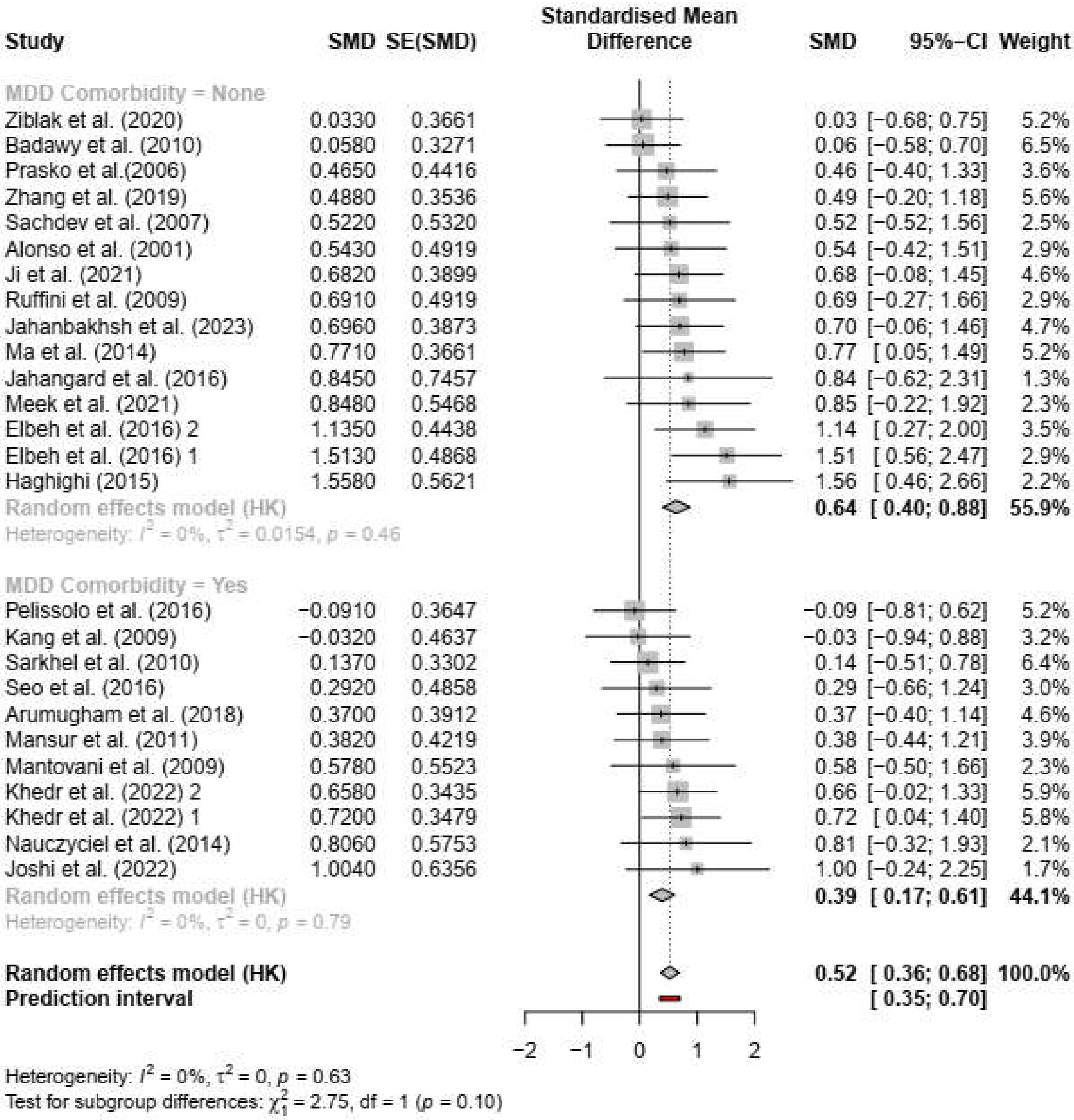

Figure S8. Effect sizes (gppc) for OCD symptoms based on the presence of MDD comorbidity with outlier studies removed.

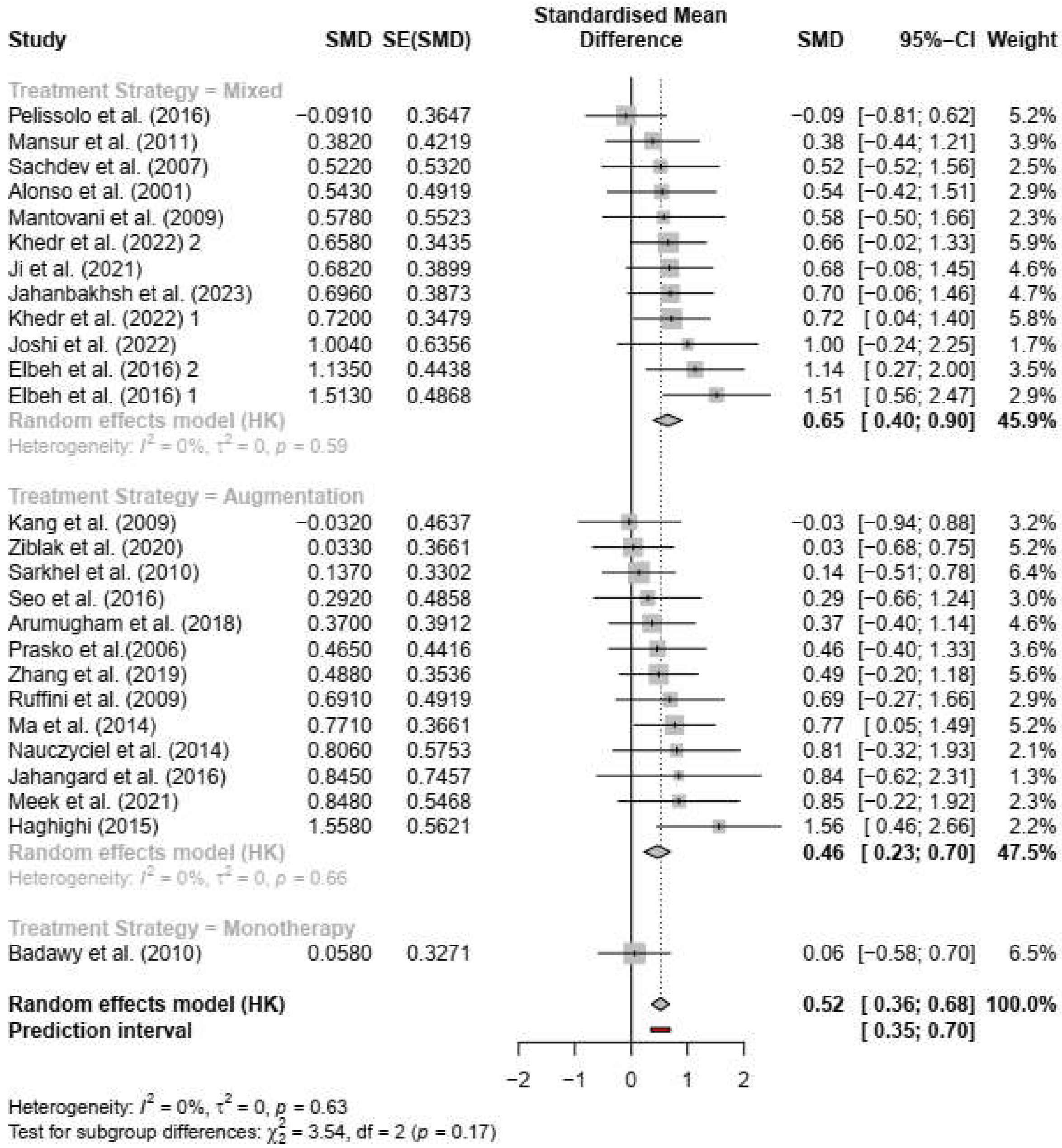

Figure S9. Effect sizes (gppc) for OCD symptoms based on the strategy of rTMS treatment rTMS with outlier studies removed

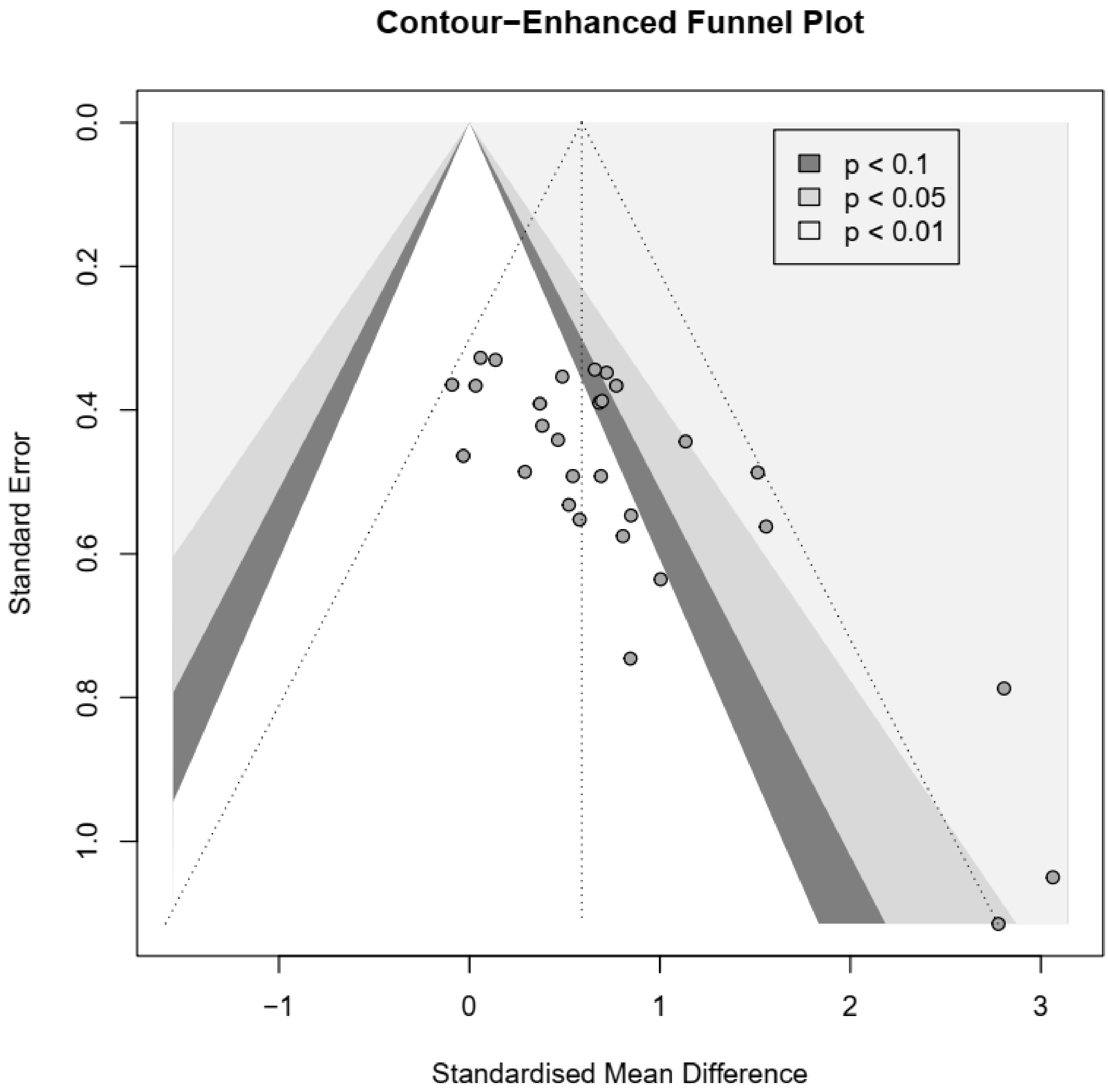

Figure S10. Contour-enhanced funnel plot for the detection of publication bias. This plot contains three shaded regions corresponding to $p < 0.1$, $p < 0.05$, $p < 0.01$. Two studies with $p < 0.01$ and one study with $p < 0.05$ have high SMDs and high standard errors which indicates the presence small study effect.

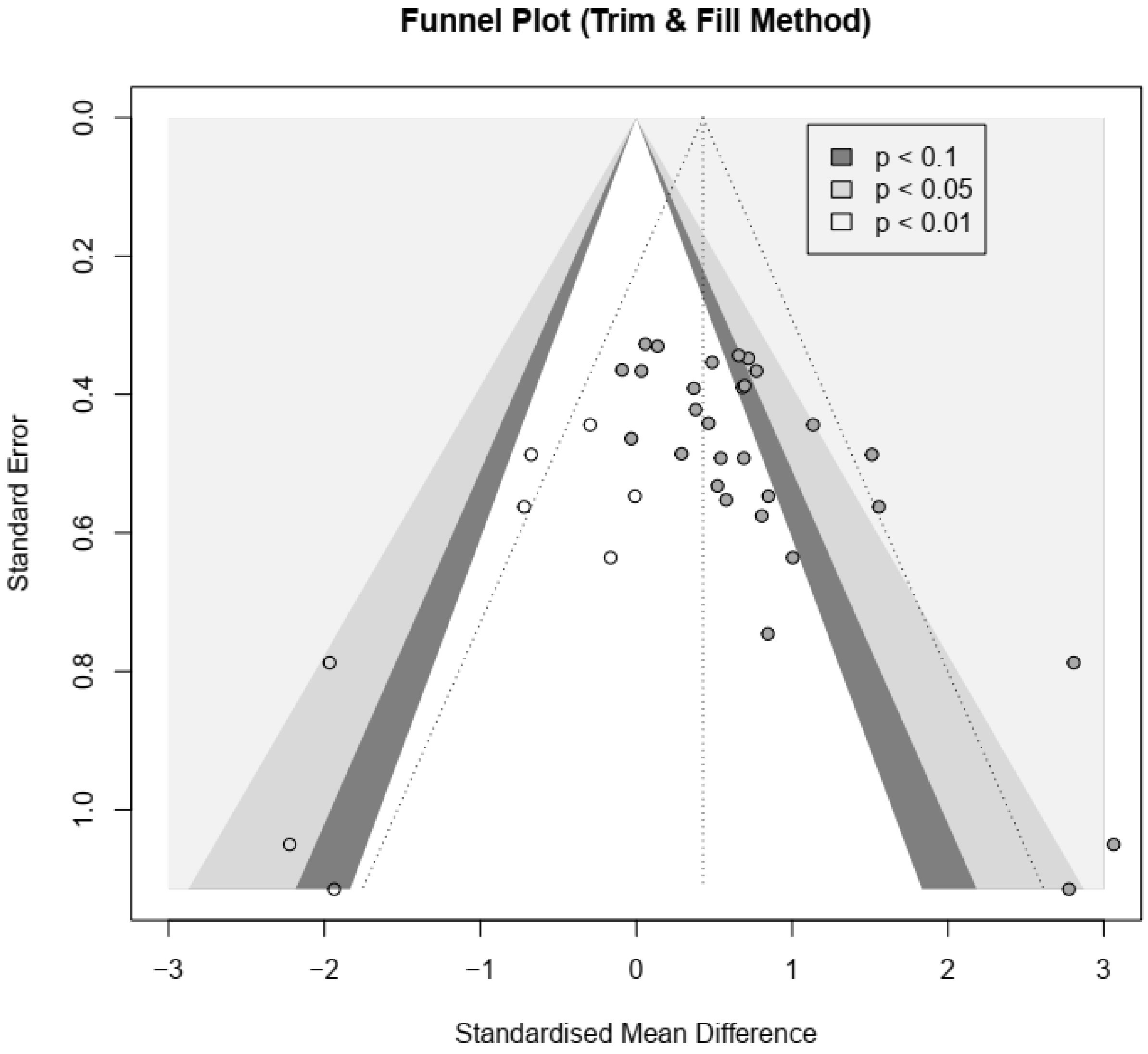

Figure S11. Funnel plot using the trim & fill method. This method added 7 studies to the existing set of studies to compensate for the asymmetry of the funnel plot that resulted in the corrected SMD of gPPC =0.34, 95% CI=0.13;0.55, p= 0.002.

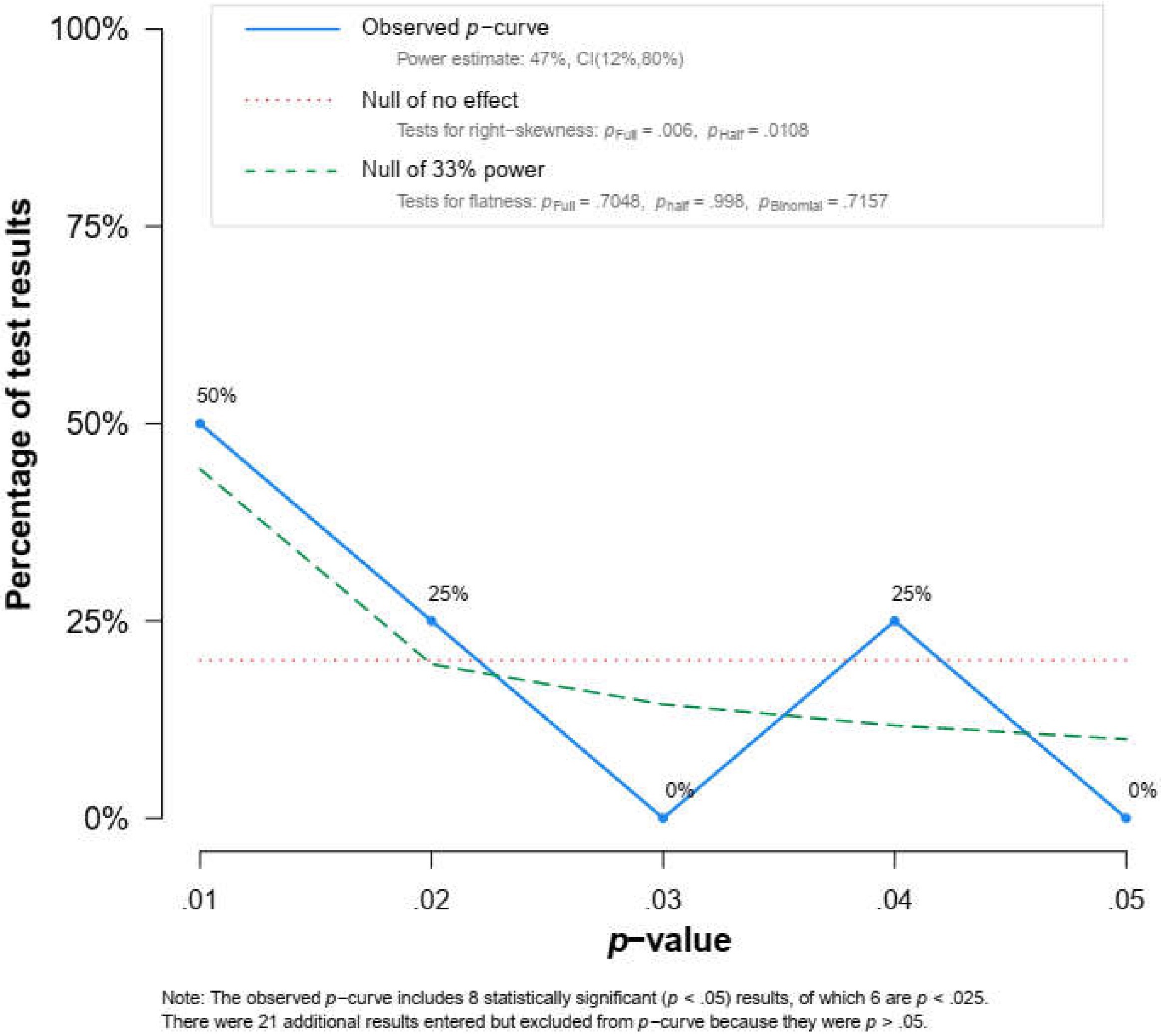

Figure S12**.** P-curve analysis of 7 statistically significant *p*-values ($p < 0.05$), of which 6 are $p < 0.025$. There were 19 additional results entered but excluded from p-curve because they were $p > .05$. The solid blue line marks the empirical distribution of the significant *p*-values among the included studies. The dotted red line shows the expected distribution of *p*-values given the null hypothesis. The dashed green line indicates the expected distribution of *p*-values given an average power of 33% for a set of studies.

Table S1: MNI Coordinates for Each ROI Used for rTMS-Induced Electrical Field Simulation

Neurocircuit	Region	Abbreviation	MNI Coordinate		
			X	Y	Z
Sensorimotor Circuit	Supplementary Motor Area	SMA	0	-1	52
	Posterior Putamen	pPut	-18	4	2
	Primary Motor Cortex	M1	37	-25	62
			-37	-25	62
	Somatosensory Cortex	--	12	-33	73
			-24	-28	70
	Insula	--	+45	3	15
			-45	5	9
	Thalamus	--	11	-12	6
			-12	-12	6
			-12	-3	13
Dorsal Cognitive Circuit	Pre- Supplementary Motor Area	Pre-SMA	+10	10	50
			-15	14	67
	Dorsolateral Prefrontal Cortex	DLPFC	+56	26	25
			-56	26	25
	Dorsomedial Prefrontal Cortex	dmPFC	-1	1	46
	Dorsal Caudate Nucleus	dCaud	+13	15	9
			-13	15	9
	Thalamus	--	11	-12	6
			-12	-12	6
			-12	-3	13
Ventral Cognitive Circuit	Inferior Frontal Gyrus	IFG	+61	21	13
			-33	23	-8
	Ventrolateral prefrontal cortex	VLPFC	42	46	0
			-32	54	-4
	Ventral Caudate Nucleus	vCaud	+10	15	0
			-10	15	0
	Thalamus	--	11	-12	6
			-12	-12	6
			-12	-3	13
Ventral Affective Circuit	Orbitofrontal cortex	OFC	34	20	-16
	Nucleus accumbens	NAcc	+9	9	8
			-9	9	8
	Thalamus	--	11	-12	6
			-12	-12	6
			-12	-3	13
Fronto-limbic Circuit	Ventromedial prefrontal cortex	VMPFC	-2	26	-2
	Amygdala	--	+22	-2	-15
			-20	-4	-15

Table S2: Induced electrical field values for each rTMS protocol corresponding to each ROI and neural circuit

Neurocircuit	Region	EF Norm Mean (SD)			
		lDPFC	rLDPFC	Pre-SMA	OFC
Whole Brain Mean (Max-Min)		13.37 (0.48-97.55)	13.08 (0.35- 97.84)	16.11 (0.81-84.54)	11.48 (0.65-79.28)
Sensorimotor Circuit	Supplementary Motor Area	5.12 (2.1)	7.11 (1.02)	28.47 (9.62)	11.03 (1.41)
	Posterior Putamen	12.87 (3.1)	6.71 (1.01)	7.56 (1.17)	1.84 (0.66)
	Primary Motor Cortex	21.1 (4.67)	21.55 (5.09)	33.11 (10.73)	8.72 (2.45)
	Somatosensory Cortex	18.17 (4.95)	17.60 (5.16)	41.03 (12.3)	8.16 (1.8)
	Insula	17.11 (3.62)	19.07 (4.94)	10.95 (2.46)	9.99 (2.21)
	Thalamus	8.95 (3.13)	6.97 (1.8)	5.65 (1.87)	3.57 (1.33)
Dorsal Cognitive Circuit	Pre- Supplementary Motor Area	10.61 (5.54)	19.48 (4.53)	41.47 (11.29)	14.21 (2.19)
	Dorsolateral Prefrontal Cortex	57.64 (11.11)	55.48 (16.37)	29.46 (6.42)	35.8 (4.86)
	Dorsomedial Prefrontal Cortex	6.17 (3.33)	6.58 (2.63)	20.43 (6.78)	10.53 (1.21)
	Dorsal Caudate Nucleus	12.65 (4.4)	8.42 (3.87)	10.34 (2.82)	5.87 (1.92)
	Thalamus	8.95 (3.13)	6.97 (1.8)	5.65 (1.87)	3.57 (1.33)

Table S3: *p*-curve analysis details. Overall, these results indicate the presence of evidential value and a true non-zero effect

		Full Curve		Half Curve		Evidential Value		
	$P_{Binomial}$	Z_{Full}	P_{Full}	Z_{Half}	P_{Half}	**Present**	**Absent**	**d**
**Right-Skewness Test**	0.145	-2.510	0.006	-2.297	0.011	Yes	No	0.66
**Flatness Test**	0.716	0.538	0.705	2.879	0.998			
